# Chronic Exposure to Malaria Is Associated with Inhibitory and Activation Markers on Atypical Memory B Cells and Marginal Zone-Like B Cells

**DOI:** 10.3389/fimmu.2017.00966

**Published:** 2017-08-21

**Authors:** Itziar Ubillos, Joseph J. Campo, Pilar Requena, Maria Ome-Kaius, Sarah Hanieh, Honor Rose, Paula Samol, Diana Barrios, Alfons Jiménez, Azucena Bardají, Ivo Mueller, Clara Menéndez, Stephen Rogerson, Gemma Moncunill, Carlota Dobaño

**Affiliations:** ^1^ISGlobal, Barcelona Ctr. Int. Health Res. (CRESIB), Hospital Clínic-Universitat de Barcelona, Barcelona, Spain; ^2^Antigen Discovery Inc., Irvine, CA, United States; ^3^Facultad de Medicina, Universidad de Granada, Granada, Spain; ^4^Papua New Guinea Institute of Medical Research, Madang, Papua New Guinea; ^5^CIBER Epidemiology and Public Health (CIBERESP), Barcelona, Spain; ^6^Walter and Eliza Hall Institute of Medical Research, Parkville, VIC, Australia; ^7^University of Melbourne, Melbourne, VIC, Australia

**Keywords:** chronic infection, malaria, tolerance, B cells, host–malaria interaction

## Abstract

In persistent infections that are accompanied by chronic immune activation, such as human immunodeficiency virus, hepatitis C virus, and malaria, there is an increased frequency of a phenotypically distinct subset of memory B cells lacking the classic memory marker CD27 and showing a reduced capacity to produce antibodies. However, critical knowledge gaps remain on specific B cell changes and immune adaptation in chronic infections. We hypothesized that expansion of atypical memory B cells (aMBCs) and reduction of activated peripheral marginal zone (MZ)-like B cells in constantly exposed individuals might be accompanied by phenotypic changes that would confer a tolerogenic profile, helping to establish tolerance to infections. To better understand malaria-associated phenotypic abnormalities on B cells, we analyzed peripheral blood mononuclear cells from 55 pregnant women living in a malaria-endemic area of Papua Nueva Guinea and 9 Spanish malaria-naïve individuals using four 11-color flow cytometry panels. We assessed the expression of markers of B cell specificity (IgG and IgM), activation (CD40, CD80, CD86, b220, TACI, and CD150), inhibition (PD1, CD95, and CD71), and migration (CCR3, CXCR3, and CD62l). We found higher frequencies of active and resting aMBC and marked reduction of MZ-like B cells, although changes in absolute cell counts could not be assessed. Highly exposed women had higher PD1^+^-, CD95^+^-, CD40^+^-, CD71^+^-, and CD80^+^-activated aMBC frequencies than non-exposed subjects. Malaria exposure increased frequencies of b220 and proapoptotic markers PD1 and CD95, and decreased expression of the activation marker TACI on MZ-like B cells. The increased frequencies of inhibitory and apoptotic markers on activated aMBCs and MZ-like B cells in malaria-exposed adults suggest an immune-homeostatic mechanism for maintaining B cell development and function while simultaneously downregulating hyperreactive B cells. This mechanism would keep the B cell activation threshold high enough to control infection but impaired enough to tolerate it, preventing systemic inflammation.

## Introduction

In persistent infections that are accompanied by chronic immune activation, such as human immunodeficiency virus (HIV), hepatitis C virus (HCV), and malaria, there is constant polyclonal proliferation of antigen-specific B cells. In such cases, homeostatic mechanisms to attenuate chronic antigenic stimulation are necessary to downregulate activation pathways and allow for host tolerance (1–3). The expression “tolerance to malaria infection,” in particular, has long been used to explain the fact that in endemic areas *Plasmodium* infection can occur without malaria disease (4).

It is accepted that in malaria and other chronic infections, sterilizing immunity rarely occurs (5, 6) and highly exposed individuals may be carriers of low-density asymptomatic infections (5, 7). In addition, there is increasing evidence that chronic *Plasmodium* parasitemia evades antibody-mediated immunity through dysregulation of CD4^+^ T cell and B cell function (5). Exposure-dependent tolerogenic antibody and cell-mediated responses likely avoid full clearance of parasitemia, a phenomenon known also as premunition (4, 7, 8).

In an effective adaptive immune response, activated B cells undergo a process of class switching recombination, somatic hypermutation (SHM) and affinity selection within the germinal center (GC) to generate long-lived plasma cells (9), memory B cells (MBCs), and protective antibodies (10). The adaptive response to an infection is a tightly controlled process in which inhibitory and proapoptotic receptors such as Fas/CD95 and PD1 (programmed death 1) play an important role in regulating cell survival (11, 12). In chronic infections like HIV (13) and malaria (14), and also in autoimmune diseases like rheumatoid arthritis (15) and systemic lupus erythematosus (16), there is upregulation of inhibitory and proapoptotic receptors on B cells coupled with increased frequency of a phenotypically distinct MBC subset lacking the classic memory marker CD27 (2, 3, 17, 18) and usually accompanied by an increase of IgD^−^CD27^+^ classical MBC (19–21). Studies of HIV- and HCV-infected individuals suggested that this CD27^−^ MBC subset may be prone to anergy and/or apoptosis, because they expressed PD1, FcRL4, FcRL5, and CD95 and had a reduced capacity to proliferate (17, 19, 22). This phenotype gave rise to the denomination of these cells as “exhausted.” A phenotypically similar subset called “atypical MBC” (aMBC) has been associated with malaria exposure (3, 18, 23–28). The role of the anergic and/or exhausted aMBC in chronic infection is still unknown.

Chronic immune activation also affects circulating IgM^+^CD19^+^CD27^+^ MBC, which frequency is greatly reduced in HIV (22) and malaria (18, 26, 29). This B cell subset is similar to marginal zone (MZ)-like B cells, found mainly in secondary lymphoid organs (30) and to a lesser extent in peripheral blood. They link innate and later-occurring adaptive responses and are key to extracellular antigen responses (31). Recent studies highlight the importance of IgM-expressing B cells in generating T-independent rapid and avid response to an infection (32–34). However, their role in chronic infection is unclear.

A common limitation of past studies is the imprecise phenotypical classification of MBC subsets. We have shown that inclusion of IgD in cytometry panels to distinguish between switched (IgD^−^) and unswitched (IgD^+^) B cells improved the specificity of MBC classification (18). Indeed, our previous work showed that a substantial frequency of CD27^−^CD21^+^, presumably naïve B cells, were actually switched MBC lacking CD27 (resting aMBC) and, conversely, that a substantial proportion of CD27^−^CD21^−^, presumably aMBC (aMBC) were actually IgD^+^ and may represent a phenotypically distinct population (18).

Here, we investigated the surface expression of multiple activation-, inhibition- and survival-associated B cell markers in peripheral blood mononuclear cells (PBMCs) from malaria-exposed and malaria-naïve donors to characterize cellular phenotypes. We hypothesized that expansion of aMBC with a tolerogenic-like phenotype and reduction of activated peripheral MZ-like B cells in constantly exposed individuals may be a complementary mechanism to downregulate continuously activated B cells while simultaneously maintaining B cell functions, helping to establish tolerance to the infection (15, 17).

## Materials and Methods

### Study Participants

The study recruited 55 malaria-exposed pregnant women from the Madang Province in Papua New Guinea (PNG) at their first antenatal clinic visit between 2008 and 2010 (Table 1) and 9 male malaria-naïve donors from Barcelona in Spain. Ten milliliters of heparinized venous blood were collected from volunteers.

**Table 1:** Characteristics of study participants by malaria exposure category.

	Non-exposed (*n* = 9)	Low-exposed (*n* = 26)	High-exposed (*n* = 29)
Age^a^	45 (31–53)	23.5 (19–36)	25 (17–38)
Body mass index	–	23.6 (12.6–29.9)	23.15 (18.6–31.6)
Gestational age (weeks)	NA	24 (18–29)	24 (15–31)
Number of pregnancies^a^	NA	2 (1–4)	1 (1–7)
Proportion with spontaneous abortion	NA	3.85	3.45
Proportion with moderate anemia (<11 mg/dL)	–	88.46	82.76
Proportion with severe anemia (<7 mg/dL)	0	3.85	6.9
Proportion with previous malaria^b^	0	23.08	13.79
Proportion with fever in the last 24 h^b^	0	7.69	3.45
Proportion with ITN use^b^	NA	42.86	57.14

*^a^Data are medians (ranges) and were self-reported*.

*^b^Self-reported*.

*ITN, insecticide-treated bed net*.

-, data not available

*NA, not applicable*.

### Ethical Approval

Written informed consent was obtained from all study participants. This study was approved by the Medical Research Advisory Committee in PNG (MRAC 08.02) and by the Hospital Clinic of Barcelona Ethics Review Committee (Comitè Ètic d’Investigació Clínica), Spain.

### Isolation of Plasma and PBMC

Plasma was separated from the cellular fraction of blood within 4 h of collection by centrifugation at 600 g for 10 min at room temperature, aliquoted and stored at −80°C. Blood cells were further fractioned in a density gradient medium (Histopaque-1077, Sigma-Aldrich) to obtain PBMCs that were frozen in fetal calf serum and 10% dimethyl sulfoxide and stored in liquid nitrogen until analysis. PNG samples were shipped to and analyzed at the Barcelona Institute for Global Health (Spain).

### Serological Classification of Intensity of Malaria Exposure

We had previously examined malaria antibody responses in 293 pregnant women from PNG (18). A Luminex microsphere technology-based assay measured IgG responses to *Plasmodium falciparum* (*Pf*) apical membrane antigen 1 (AMA1), merozoite surface protein 1 (MSP-1_19_), and region II of the 175 kDa erythrocyte binding protein (EBA175); as well as *Plasmodium vivax* (*Pv*) merozoite surface protein 1 (MSP-1_19_) and Duffy binding protein, all expressed in *Escherichia coli*. Antibody levels were expressed as median fluorescence intensity (MFI). For an in-depth B cell analysis, we selected a subset of individuals with diverse antibody profiles. We classified malaria exposure as high or low according to the magnitude and breadth of antibody levels. We used serology as an indirect measure of both recent and past cumulative exposure to *Plasmodium* to build a classifier for exposure levels. It is well established in malaria that with cumulative exposure there is an increase in the breadth of response to *Pf* and *Pv* antibody levels (35–40). For magnitude, IgG responses were divided in tertiles and ranked from 1 (low) to 3 (high) for each of the five antigens. For breadth, combined responses ranking from 5 to 9 were classified as low exposure, and those ranking from 10 to 15 as high exposure (Figure S1 in Supplementary Material). We then randomly selected 55 among the 293 malaria-exposed women; 29 were highly exposed and 26 had low exposure.

### Phenotyping and Gating Strategy

Peripheral blood mononuclear cells were thawed, and their viability measured on a Guava^®^ Cytometer using Guava ViaCount Reagent (Millipore, Madrid, Spain). All samples had viabilities >60% and were used for the assays. All antibodies and reagents were purchased from BD Biosciences (Madrid, Spain), unless otherwise indicated. For compensation controls, BD Comp Beads were used. Cells and beads were acquired on a BD LSR II Fortessa cytometer.

One million PBMCs per sample were used for B cell staining. Cell suspensions were stained with LIVE/DEAD^®^ Fixable Aqua Dead Cell Stain Kit (Invitrogen, Madrid, Spain), washed and blocked with PBS-BSA 0.05%. After washing, cells were stained in four multiparametric panels having a core panel with the following antibodies: anti-CD3 Horizon v500 (clone UCHT1), anti-CD14 Horizon v500 (clone M5e2), anti-CD16 Horizon v500 (clone 3G8), anti-CD19 PE/CF594 (clone HIB19), anti-IgD allophycocyanin/H7 (clone IA6-2), anti-CD27 allophycocyanin (clone M-T271), anti-surface IgD-APC/H7 (clone IA6-2), anti-CD21 FITC (Beckman Coulter, clone BL13), anti-CD38 PerCP (clone HIT2), and anti-CD10 BV421 (BioLegend, clone HI10a). The additional sets of antibodies added to the core panel were as follows: (1) anti-surface IgM PE (clone G20-127), anti-sIgG PE/Cy7 (clone G18-145), rat anti-mouse b220 Alexa Fluor 700 (clone RA3-6B2), and anti-PD1 BV605 (clone EH12.2H7); (2) anti-CD150 PE (clone A12), anti-CD95 PE/Cy7 (clone DX2), anti-CD40 Alexa Fluor 700 (clone 5C3), and anti-TACI BV605 (BioLegend, clone 1A1); (3) anti-CCR3 PE (clone 5E8), anti-CD71 PE/Cy7 (eBioscience, clone OKT9), anti-CXCR3 Alexa Fluor 700 (clone 1C6), and anti-CD62L BV6054 (clone DREG-56); and (4) anti-CD80 Alexa Fluor 700 (clone L307.4) and anti-CD86 PE/Cy7 (clone 2331).

**Figure 1:**
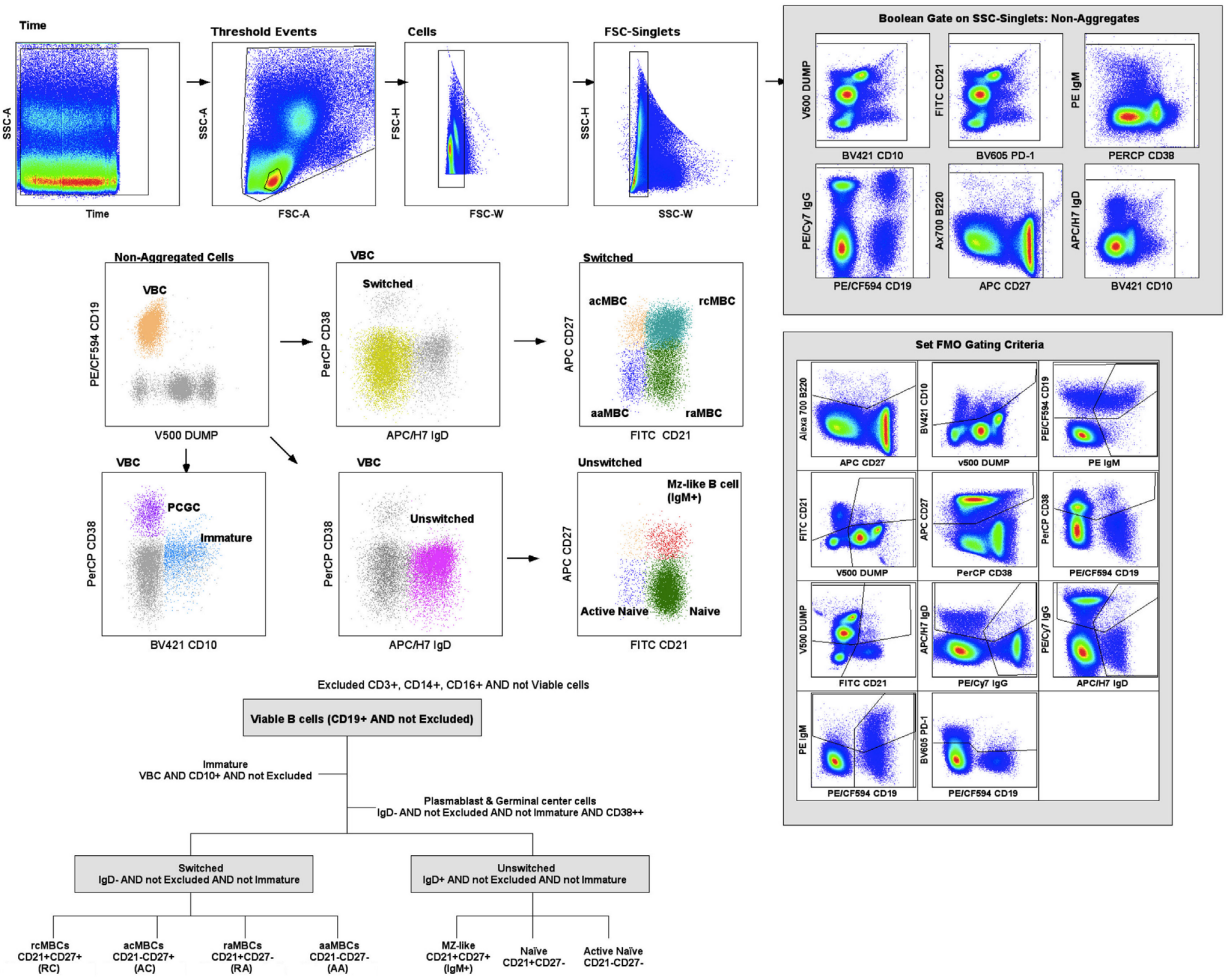
Gating strategy. Cell acquisition was performed on a BD LSR II Fortessa cytometer and data analyzed on Flow Jo (Tree Star). B cell classification was performed based on Boolean gates. Excluded population (CD3^+^CD14^+^CD16^+^ and viability marker); viable B cells (VBC) (CD19^+^ AND NOT Excluded); immature B cells (VBC AND CD10^+^); plasmablasts and germinal center cells (VBC AND NOT Immature AND IgD^−^ AND CD38^high^); switched (VBC AND NOT Immature AND NOT CD38^high^ AND IgD^−^); AC indicates active classical memory B cells (MBCs) (Switched AND CD21^−^CD27^+^); RC, resting classical MBCs (Switched AND CD21^+^CD27^+^); AA, active atypical MBCs (Switched AND CD21^−^CD27^−^); RA, resting atypical MBCs (Switched AND CD21^+^CD27^−^); unswitched (VBC AND NOT Immature AND NOT CD38^high^ AND IgD^+^); naïve (Unswitched AND CD21^+^CD27^−^); active naïve (Unswitched AND CD21^−^CD27^−^) and marginal zone (MZ)-like B cells (Unswitched AND CD21^+^CD27^+^ AND IgM^+^).

Fluorescence-minus-one controls were used for gating of positive events (Figure S2 in Supplementary Material). Samples were stained containing all the antibodies of the panel except one marker. These samples were used to determine the negative population for each staining experiment. Figure 1 shows the gating strategy used for this study. Lymphocytes were gated using a time event gate, excluding duplets. Live B cells were displayed according to CD19^+^ expression and a dump channel containing a viability marker, CD3, CD14, and CD16. Mature viable B cells (VBC) were gated through a Boolean gate containing live B cells, CD19^+^, and not CD10^+^ cells. Immature B cells contained CD19^+^, CD10^+^, and not CD38^++^. VBC were further divided into switched (IgD^−^) or unswitched (IgD^+^) populations, and plasmablast and germinal center cells (PCGC) (IgD^−^CD38^++^) (Figure 1). Switched and unswitched populations were displayed according to their expression of CD21 and CD27, and MBC classified as active (CD21^−^) and resting (CD21^+^). Unswitched (IgD^+^) population was divided as naïve B cells, MZ-like MBC and active naïve. The switched (IgD^−^) population was classified as active classical MBC (acMBC), resting classical MBC (rcMBC), active atypical MBC (aaMBC), and resting atypical MBC (raMBC) (Figure 1). Every B cell subset was expressed as percentage of total VBC. Shifts in one B cell subset were accompanied by shifts in one or more other B cells subsets. Gating strategy and data analysis was performed on FlowJo software version 9.2 (Tree Star).

### Statistical Analysis

Percentage cell population and frequency of marker expression were compared between three malaria exposure groups, non-exposed, low- and high-exposed individuals, using Kruskal–Wallis test plus Dunn’s pairwise *post hoc* test. Differences between exposed and non-exposed individuals were assessed with the Wilcoxon signed-rank test and adjusted for multiple comparison by the Benjamini and Hochberg method (41). To compare aMBC and MZ-like B cell frequencies, and marker MFI against the rest of B cell subsets, we used the Friedman test, with Dunn’s *post hoc* test. Analyses and figures were performed using Stata (StataCorp, 2014, Statistical Software, College Station, TX, USA: StataCorp LP) and GraphPad Prism (La Jolla, CA, USA).

## Results

### Malaria Exposure Is Associated with Differences in Frequencies of Circulating B Cell Subpopulations

**Figure 2:**
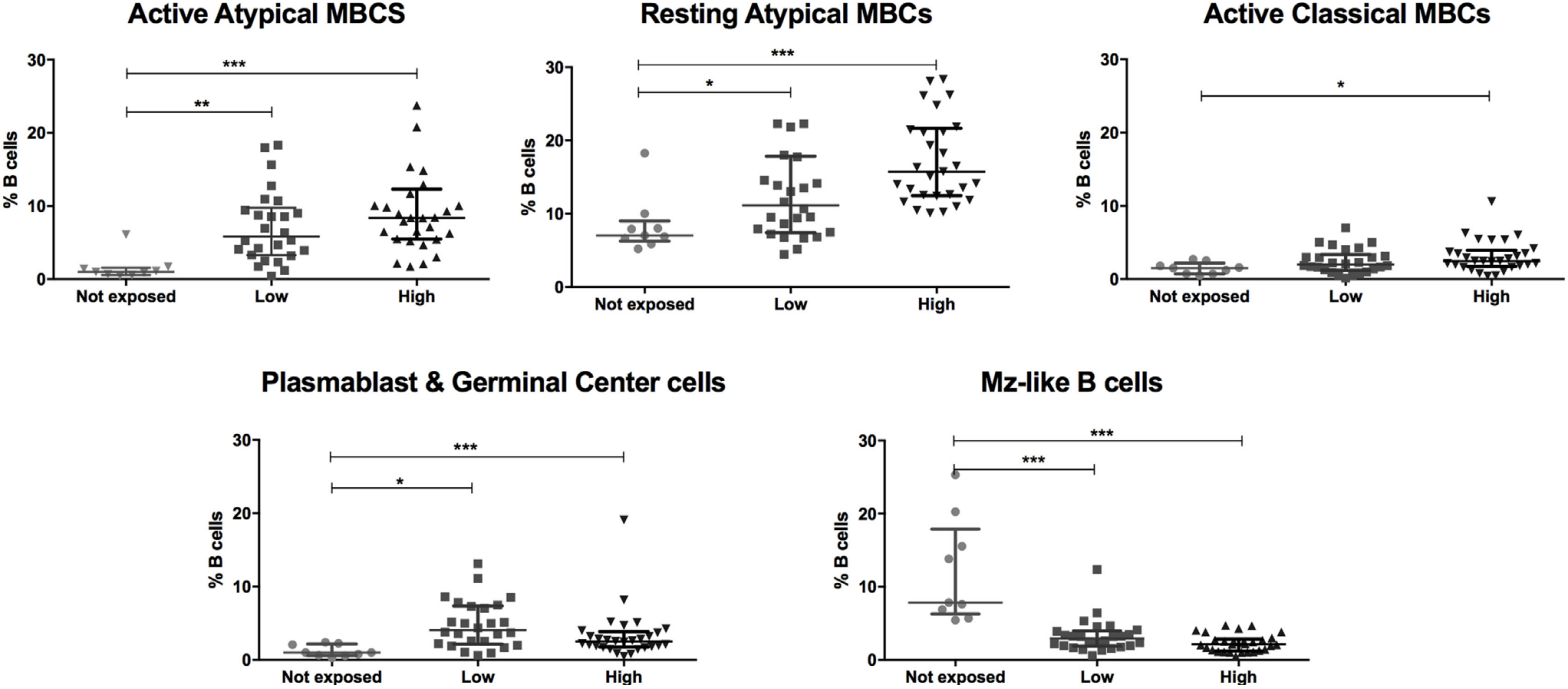
Malaria exposure is associated with changes in the frequencies of different B cell subsets. Beeswarm plots show B cell subset frequencies in the groups categorized by malaria exposure. No exposure (Not exposed; *n* = 9), low exposure (Low; *n* = 26), and high exposure (High; *n* = 29). Lines and whiskers represent median and interquartile range, respectively. Differences were assessed with Kruskal–Wallis test plus Dunn’s *post hoc* test comparing each exposure category (*p ≤ 0.05, **p ≤ 0.01, ***p ≤ 0.001).

Baseline population characteristics are described in Table 1. B cell subsets were characterized (Figure 1) and compared between exposed and non-exposed controls (Table 2), specifically 9 malaria non-exposed, 26 malaria low-exposed, and 29 malaria high-exposed pregnant women. We analyzed the proportions of the following cells: aaMBCs (CD3^−^CD14^−^CD16^−^CD19^+^CD10^−^IgD^−^CD21^−^CD27^−^); raMBCs (CD3^−^CD14^−^CD16^−^CD19^+^CD10^−^IgD^−^CD21^+^CD27^−^); acMBCs (CD3^−^CD14^−^CD16^−^CD19^+^CD10^−^IgD^−^CD21^−^CD27^+^); rcMBCs (CD3^−^CD14^−^CD16^−^CD19^+^CD10^−^IgD^−^CD21^+^CD27^+^); PCGC (CD3^−^CD14^−^CD16^−^CD19^+^CD10^−^IgD^−^CD38^high^); naïve (CD3^−^CD14^−^CD16^−^CD19^+^CD10^−^IgD^+^CD21^+^CD27^−^); active naïve (CD3^−^CD14^−^CD16^−^CD19^+^CD10^−^IgD^+^CD21^−^CD27^−^); MZ-like B cells (CD3^−^CD14^−^CD16^−^CD19^+^CD10^−^IgD^+^CD21^+^CD27^+^IgM^+^); and immature B cells (CD3^−^CD14^−^CD16^−^CD19^+^CD10^+^). B cell classification was performed based on previous phenotyping experiments (19, 42). The classification of plasmablast and GC (or GC-like) cells follows the Bm1–5 gating strategy using IgD and CD38 (42); it is worth noting that the PCGC subpopulation represents a small fraction of circulating B cells, and that some pre-GC and recent GC B cells may be caught in the peripheral blood. Eight individuals had a *Pf* infection, measured by PCR, three individuals in the high-exposure and five individuals in the low-exposure groups. We found no significant differences in B cell subset frequencies and proportions of marker expression in infected versus non-infected individuals, as shown before for aMBC or MZ-like B cells in this cohort (18) and as reported by others (3, 43) (Table S1 in Supplementary Material). Thus, individuals with infection were included in the overall analysis. As previously shown in the literature, frequencies of peripheral blood aaMBC, raMBC, acMBC, and PCGC were increased in exposed individuals (Table 2). We also confirmed the strong decline in the proportion of circulating MZ-like B cells with malaria exposure. Overall, we did not find statistically significant differences in the B cell subset proportions between high- and low-exposed individuals. However, for aaMBC, raMBCs, and acMBC, the differences were greater when non-exposed were compared to individuals with high exposure than to those with low exposure (Figure 2). Based on these results, we focused our study on switched (atypical and classical) MBC and MZ-like B cells.

**Table 2:** B cell subpopulation percentages calculated as proportion of total viable B cells in malaria non-exposed (*n* = 9) and malaria-exposed (*n* = 55) individuals and 95% confidence interval.

B cell subsets	Not exposed		Exposed		p*

	%	95% CI	%	95% CI	
Naïve	39.66	31.7–47.6	31.67	28.2–35.2	0.11
Active naïve	1.78	0.0–3.5	3.25	2.7–3.8	0.005
MZ-like B cells	12.05	6.6–17.5	2.76	2.3–3.3	<0.001
Plasmablast and germinal center	1.21	0.6–1.8	8.98	7.2–10.7	0.023
Immature	7.8	5.3–10.3	4.04	3.1–5.0	0.962
Active atypical MBCs	1.54	0.2–2.9	9.08	7–11.2	<0.001
Resting atypical MBCs	8.42	5.4–11.5	15.75	12.7–17.8	0.002
Active classical MBCs	1.48	0.9–2.1	2.8	2.3–3.3	0.041
Resting classical MBCs	20.27	16.5–24.1	17.72	15.6–19.8	0.21

*p-Values adjusted for multiple testing by Benjamini and Hochberg method.

*MZ-like B cells, marginal zone-like B cells; MBC, memory B cell*.

### Malaria Exposure Alters Atypical and Classical MBC Phenotypes

**Figure 3:**
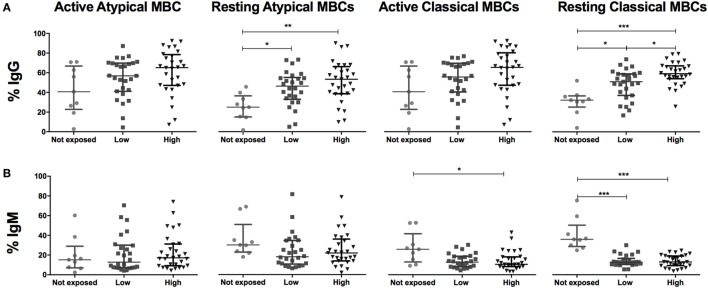
Malaria exposure and surface expression of IgG and IgM in switched memory B cells (MBCs). Beeswarm plots show **(A)** IgG and **(B)** IgM expression on MBCs subsets across exposure categories: Not exposed (*n* = 9), Low (*n* = 26), and High (*n* = 29) exposure. Active atypical MBCs (CD19^+^CD10^−^IgD^−^CD21^−^CD27^−^); resting atypical MBCs (CD19^+^CD10^−^IgD^−^CD21^+^CD27^−^); active classical MBCs (CD19^+^CD10^−^IgD^−^CD21^−^CD27^+^); and resting classical MBCs (CD19^+^CD10^−^IgD^−^CD21^+^CD27^+^). Lines and whiskers represent median and interquartile range, respectively. Differences were assessed with Kruskal–Wallis test plus Dunn’s *post hoc* test comparing each exposure category (*p < 0.05, **p < 0.01, ***p < 0.001).

We compared frequencies of B cell subsets expressing markers of activation and co-stimulation (IgG, IgM, b220, CD40, CD150, CD80, and CD86), inhibition (PD1, CD95, and TACI) and migration (CD62L, CXCR3, CCR3, and CD71) between malaria-exposed and non-exposed individuals (Table 3; Table S2 in Supplementary Material) and between different categories of exposure and non-exposed (Figure 3). Non-statistically significant differences were detected between low-exposed and high-exposed pregnant women in IgG^+^ aaMBC or IgG^+^ acMBC frequencies, but IgG^+^ raMBC and IgG^+^ rcMBC expression was increased in highly exposed individuals (Figure 3A). IgM^+^ aaMBC and IgM^+^ raMBCs were not affected by exposure. However, IgM-expressing acMBC and rcMBCs had a marked reduction in frequency with increased malaria exposure (Figure 3B), as seen in previous studies on HIV-infected individuals (2).

**Table 3:** Phenotypic marker frequencies in malaria-exposed and non-exposed individuals.

	aaMBC, mean (95% CI)			raMBC, mean (95% CI)			MZ-like B cells, mean (95% CI)		
	Not exposed	Exposed	p*	Not exposed	Exposed	p*	Not exposed	Exposed	p*
IgG	42.08 (23.3–60.8)	57.6 (51.7–63.4)	0.164	25.26 (14.8–35.6)	47.8 (42.5–53)	0.015	1.69 (0.4–2.9)	4.09 (2.4–5.7)	0.224
IgM	19.92 (5.8–34)	22.3 (17.3–27.2)	0.744	36.38 (22–50.7)	24.7 (20–29.3)	0.113	NA	NA	NA
b220	34.08 (13.4–54.7)	14.93 (10.6–19.2)	0.149	23.54 (9.9–37.1)	16.07 (11.8–20.2)	0.373	8.49 (3.7–13.2)	14.49 (12.2–16.7)	0.7
PD1	5.64 (1.2–10)	19.74 (15.6–23.8)	0.027	0.49 (0.2–0.7)	1.69 (1–2.3)	0.155	1.07 (0.1–2.1)	4.27 (2.3–6.2)	0.052
CD40	49.98 (31.6–68.3)	71.63 (65.6–77.6)	0.065	90.01 (80–99.9)	95.39 (93.8–96.8)	0.373	82.63 (71.1–94.1)	88.7 (86.2– 91.3)	0.299
CD95	44.13 (25.5–62.7)	83.84 (80.7–86.9)	0.002	21.56 (7–36.1)	63.73 (58.3–69)	0.002	29.67 (8.3–51)	45.77 (38.5–53)	0.109
TACI	74.42 (64.1–84.6)	66.1 (59.6–72.5)	0.665	76.55 (63.6–89.4)	70.68 (64.7–76.5)	0.697	90.1 (79.5–99.6)	74.76 (69.1–80.3)	0.047
CD150	17.04 (6.2–27.8)	14.62 (11.1–18)	0.702	10.31 (−1.6 to 22.3)	13.47 (9.9–16.9)	0.163	22.46 (−2.4 to 47.3)	36.49 (28.5–44.4)	0.109
CCR3	18.98 (5.1–32.8)	17.17 (11.4–22.8)	0.695	12.34 (−10.7 to 35.4)	14.18 (7.2–21.1)	0.372	13.11 (−8.5 to 34.8)	14.24 (7.6–20.8)	0.837
CXCR3	1.23 (0–2.5)	2.95 (1.6–4.2)	0.162	2.8 (0.8–4.8)	4.42 (1.4–7.4)	0.88	1.99 (0.4–3.5)	4.27 (1.7–6.7)	0.403
CD71	27.75 (14.2–41.2)	47.48 (41.4–53.5)	0.056	22.96 (1.9–43.9)	35.01 (28.5–41.5)	0.127	10.79 (−6 to 27.6)	15.06 (9.6–20.5)	0.164
CD62L	14.87 (10.6–19.1)	15.43 (12.1–18.6)	0.62	23.16 (13.4–32.8)	31.65 (26.7–36.5)	0.332	29.8 (15.9–43.7)	31.87 (26.8–36.8)	0.855
CD80	3.91 (1.4–6.3)	9.29 (7–11.4)	0.043	4.81 (2.2–7.3)	10.19 (8.3–12)	0.027	1.35 (0.8–1.8)	2.85 (2.2–3.4)	0.128
CD86	32.67 (18–47.3)	35.5 (30.8–40.1)	0.822	7.24 (−0.7 to 15.2)	19.57 (15.1–23.9)	0.009	9.19 (0.1–18.2)	12.39 (8.4–16.2)	0.162

*Percentages of B cell subsets expressing specific marker were calculated as proportions of total B cell subset*.

*p-Values adjusted for multiple testing by Benjamini and Hochberg method, comparing malaria-exposed and non-exposed individuals.

*NA, not applicable; MBC, memory B cell; MZ, marginal zone; aaMBC, active atypical MBC; raMBC, resting atypical MBC*.

*aaMBCs (CD19^+^CD10^−^IgD^−^CD21^−^CD27^−^), raMBCs (CD19^+^CD10^−^IgD^−^CD21^+^CD27^−^), and MZ-like B cells (CD19^+^CD10^−^IgD^+^CD21^+^CD27^+^IgM^+^)*.

**Figure 4:**
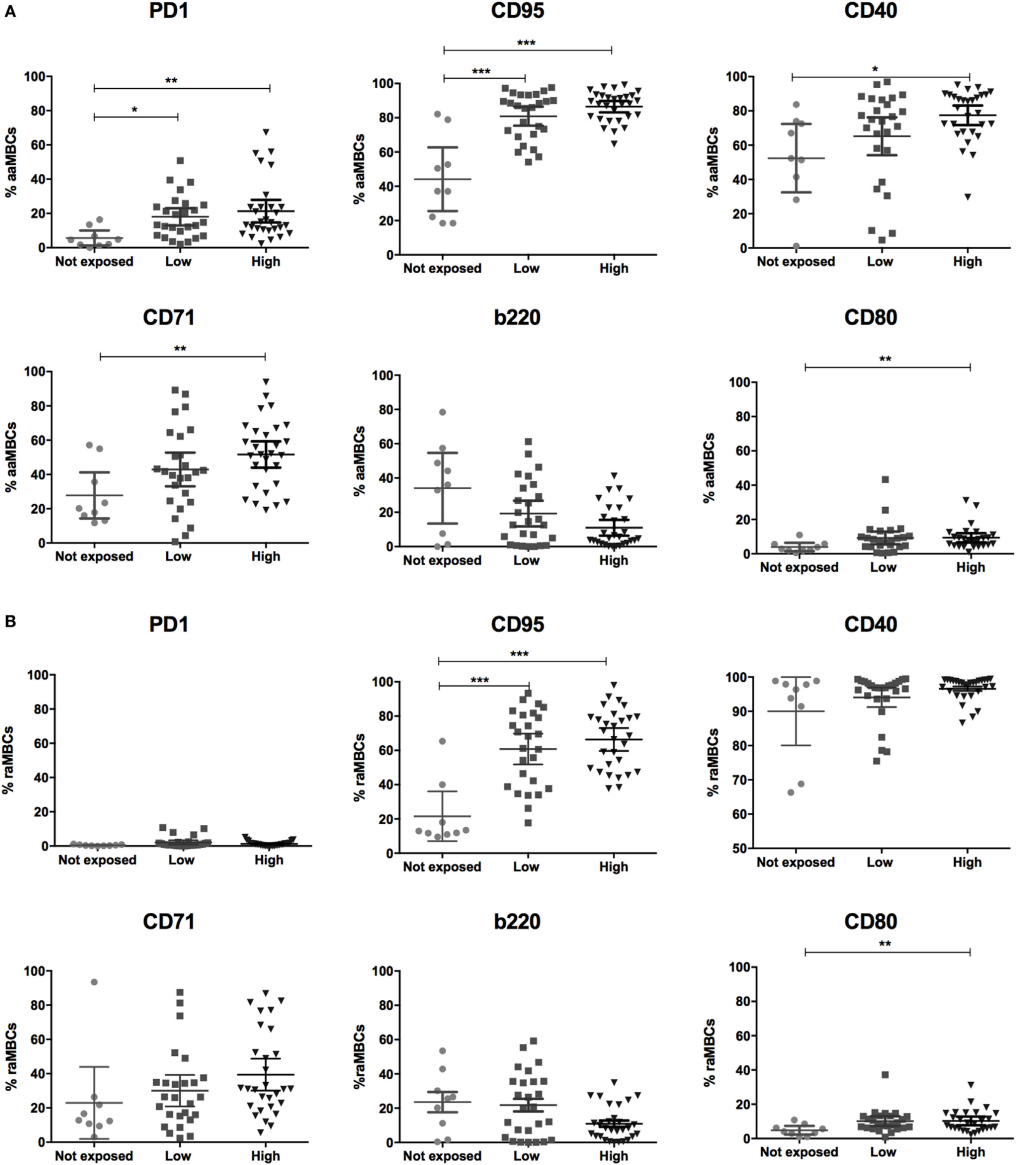
Malaria exposure is associated with changes in atypical memory B cells (MBCs) phenotypes. Graphs show the frequencies of PD1, CD95, CD40, CD71, and b220-expressing **(A)** active atypical MBCs (aaMBCs, CD19^+^CD10^−^IgD^−^CD2^−^CD27^−^) and **(B)** resting atypical MBCs (rcMBCs, CD19^+^CD10^−^IgD^−^CD21^+^CD27^−^) among malaria exposure categories: Not exposed (*n* = 9), Low (*n* = 26), and High (*n* = 29) exposure. Beeswarm plots with lines representing median and interquartile range, respectively. Differences were assessed with Kruskal–Wallis test plus Dunn’s *post hoc* test comparing each exposure category (*p < 0.05, **p < 0.01, ***p < 0.001).

PD1, a marker of T cell activation and exhaustion, plays important roles in negative regulation of T cell responses and maintenance of peripheral tolerance in malaria infection (25). Frequencies of PD1^+^ aaMBCS were significantly increased in exposed individuals (Table 3; Figure S3 in Supplementary Material), with higher frequencies of PD1^+^ aaMBCS in low and high-exposed compared to non-exposed volunteers (Figure 4A). No differences in the expression of this marker were observed for the other switched B cell subsets. Increased expression of Fas/CD95 by circulating B cells has been related to MBC activation (16), described in chronic antigen exposure (15, 16, 19) and associated with induction of apoptosis (12). Frequencies of CD95^+^-expressing aaMBCs, raMBCs, acMBCs, and rcMBCs were increased in exposed individuals compared to non-exposed (Table 3; Table S2 in Supplementary Material). However, no statistically significant differences were found between high and low malaria-exposed groups (Figure 4; Figure S3 in Supplementary Material).

B cell activation by a T-dependent signaling pathway involves the interaction with co-stimulatory molecules: CD28 on T cells, CD80 and CD86 on B cells. CD80 also binds to PD1 ligand (PD1L) providing a negative signal for proliferation and IgG secretion of normal B cells. In contrast, CD86 enhances B cell activity (44). Interactions of PD1L with its two ligands, PD1 and CD80, on T cells play a key role in controlling T cell activation, proliferation, anergy and apoptosis (45).

Triggering of CD80 specifically inhibits B cell proliferation, whereas CD86 enhances B cell activity (53). Malaria-exposed women had increased frequencies of CD80^+^ active and raMBCs and CD86^+^ resting atypical and classical MBC compared to non-exposed (Table 3; Table S2 in Supplementary Material).

CD40 signaling is required for GC formation but, paradoxically, strong CD40 signaling causes B cells to become GC-independent MBCs. It also provides short-term rescue signals to avoid CD95-induced death by upregulating antiapoptotic proteins (32, 46). We observed higher frequencies of CD40^+^ aaMBCs and CD40^+^ acMBCs in malaria-exposed compared to non-exposed individuals, with a tendency of increased frequencies with higher exposure. No effect of malaria exposure was observed on CD40^+^ resting classical or atypical MBCs (Figure 4; Figure S4 in Supplementary Material).

Transferrin receptor (CD71) is a cell surface molecule that regulates uptake of iron-bound transferrin by receptor-mediated endocytosis, is expressed in dividing cells and is a marker of B cell endocytosis (18, 47, 48–50). Highly exposed individuals had increased frequencies of CD71^+^ aaMBCs compared to non-exposed individuals (Figure 4A), but no other association with malaria exposure was found for CD71 expression in the remaining switched B cell subsets (Table S2 in Supplementary Material).

B cells downregulate expression of b220 (a glycoform of CD45R) after or during migration from follicular mantle zones to GCs and prior to reentering circulation (51). We found no statistically significant differences in the frequency of b220^+^ cells between exposed and non-exposed individuals for any of the B cell subsets (Table S2 in Supplementary Material; Table 3).

### Malaria Exposure Is Associated with Upregulation of Inhibitory Markers on Peripheral MZ-Like and Active Naïve B Cells

**Figure 5:**
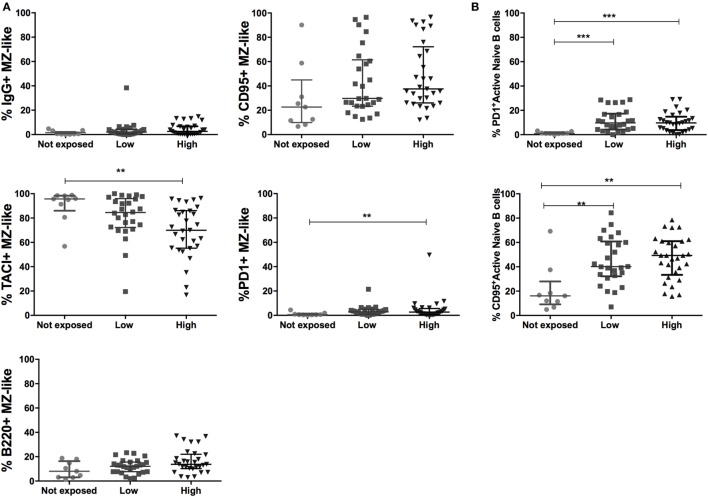
Malaria exposure and upregulation in inhibitory markers on marginal zone (MZ)-like and active naïve B cells. **(A)** MZ-like B cells (CD10^−^IgD^+^CD21^+^CD27^+^IgM^+^) expressing IgG, TACI, b220, CD95, and PD1 and **(B)** active naïve B cell expressing CD95 and PD1 in Non- (*n* = 9), Low- (*n* = 26), and High-exposed (*n* = 29) individuals. Beeswarm plots with lines representing median and interquartile range, respectively. Differences were assessed with Kruskal–Wallis test plus Dunn’s *post hoc* test comparing each exposure category (*p < 0.05, **p < 0.01, ***p < 0.001).

To investigate changes that drive the intense depletion of circulating MZ-like B cells in individuals chronically exposed to malaria, we analyzed the surface expression of activation, inhibition, and migration markers on peripheral MZ-like B cells across exposure categories. PD1^+^ MZ-like B cells were more frequent in individuals with the highest malaria exposure level compared to non-exposed (Figure 5A). We also found increased proportions of IgG^+^, CD95^+^, and b220^+^ MZ-like B cells with malaria exposure, although differences did not reach statistical significance (Figure 5A). The trans-membrane activator calcium modulator and cyclophilin ligand interactor (TACI), a B cell activation factor (BAFF) receptor, promotes the differentiation of B cells into plasma cells (41, 42). We found decreased proportions of TACI^+^ MZ-like B cells in the highly exposed group compared to the non-exposed one (Figure 5A). In summary, malaria exposure reduced circulating MZ-like B cells, increased expression of b220 and proapoptotic markers PD1 and CD95, and decreased expression of the activation marker TACI. Finally, we also found increased frequencies of CD95 and PD1 expression on active naïve B cells (Figure 5B; Table S2 in Supplementary Material).

### aaMBCs Express More Inhibitory Markers Compared to Other B Cell Subsets in Malaria-Exposed Individuals

**Figure 6:**
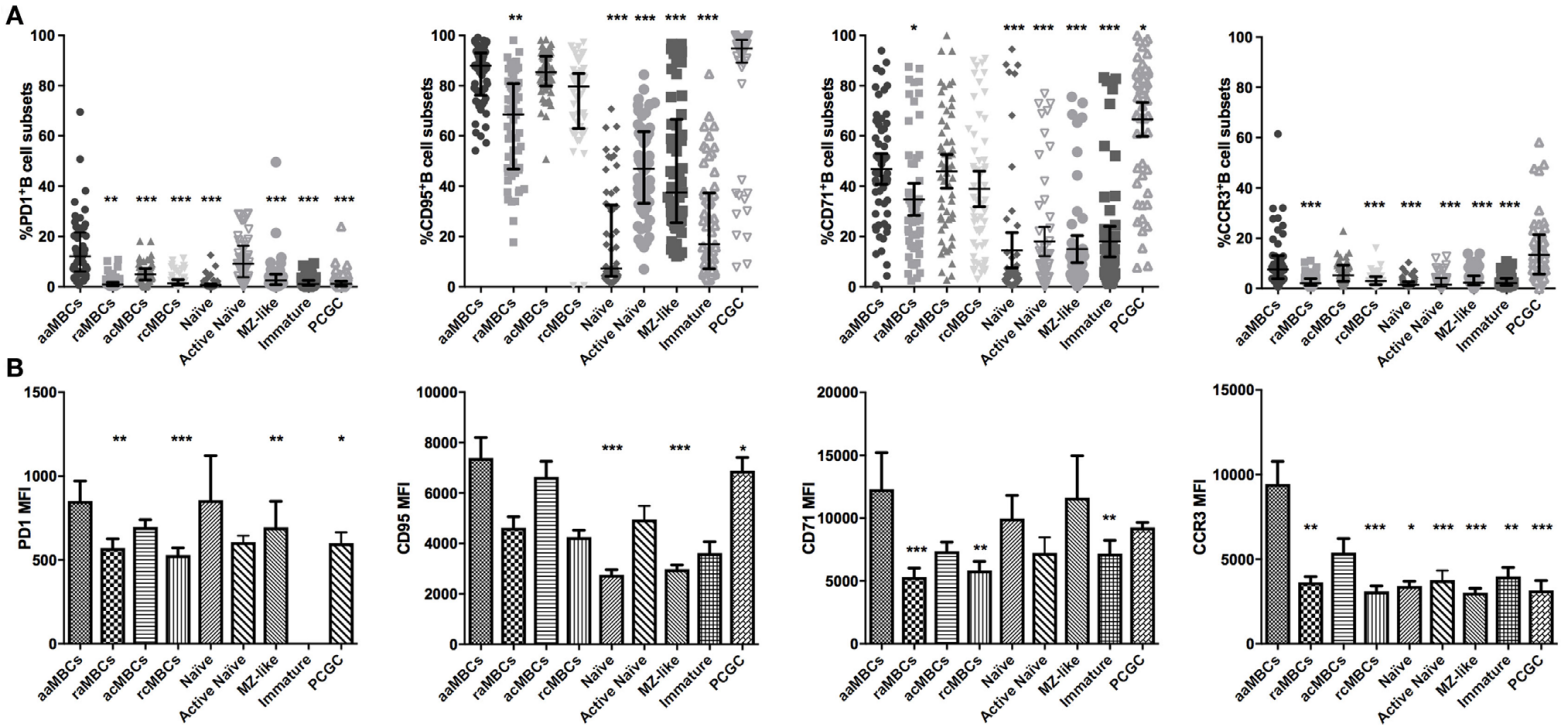
Expression of PD1, CD95, CD71, and CCR3 in B cell subpopulations in malaria-exposed individuals. **(A)** Frequencies (%) expressed median and interquartile range, respectively, and **(B)** magnitude of expression, each bar represents the SE about the mean. Differences were assessed with the Friedman test plus Dunn’s *post hoc* test, comparing active atypical memory B cells (aaMBCs) versus every other B cell subset (*N* = 55; *p < 0.05, **p < 0.01, ***p < 0.001). MBC, memory B cell; raMBC, resting atypical MBC; acMBC, active classical MBC; rcMBC, resting classical MBC; PCGC, plasmablast and germinal center cells.

We described the phenotypic profile of atypical aMBC and other B cell subsets in malaria-exposed women to assess the effect of chronic exposure. For this objective, we included only malaria-exposed individuals in the analysis. As expected, aaMBC and acMBCs had the highest frequencies of IgG^+^ cells within the switched compartment, and IgM was highly expressed in unswitched B cells subsets (Table 3; Table S2 in Supplementary Material). PD1 was expressed on aaMBCs more than in any other B cell subset, both expressed as percentage of positive cells and MFI values (Figure 6). Frequencies and levels of expression of CD95 were similar between aaMBCs, acMBCs, rcMBS, and PCGC subsets, and higher compared to the rest of the subsets (Table 3; Figure 6A). PCGC had the highest percentage of CD71^+^ cells, followed by the other switched MBC (Table S3 in Supplementary Material; Figure 6A), but the highest magnitude of CD71 expression was found on aaMBCs (Figure 6B). aaMBCs and raMBCs had the highest CD80 expression, whereas the unswitched, MZ-like, naïve, and active naïve B cells had the lowest frequency (Table 3; Table S2 in Supplementary Material).

In addition, as recent studies on the origin of aaMBCs suggested that aaMBCs and acMBCs may come from the same precursor (14, 57), we analyzed paired expression of our markers in aaMBC and acMBC to assess hypothetical differences in marker expression. We found that aaMBC had higher frequencies of PD1^+^, b220^+^, IgM^+^, and CD86^+^ cells and lower proportions of CD62L^+^cells than acMBC (Figure S5 in Supplementary Material).

**Figure 7:**
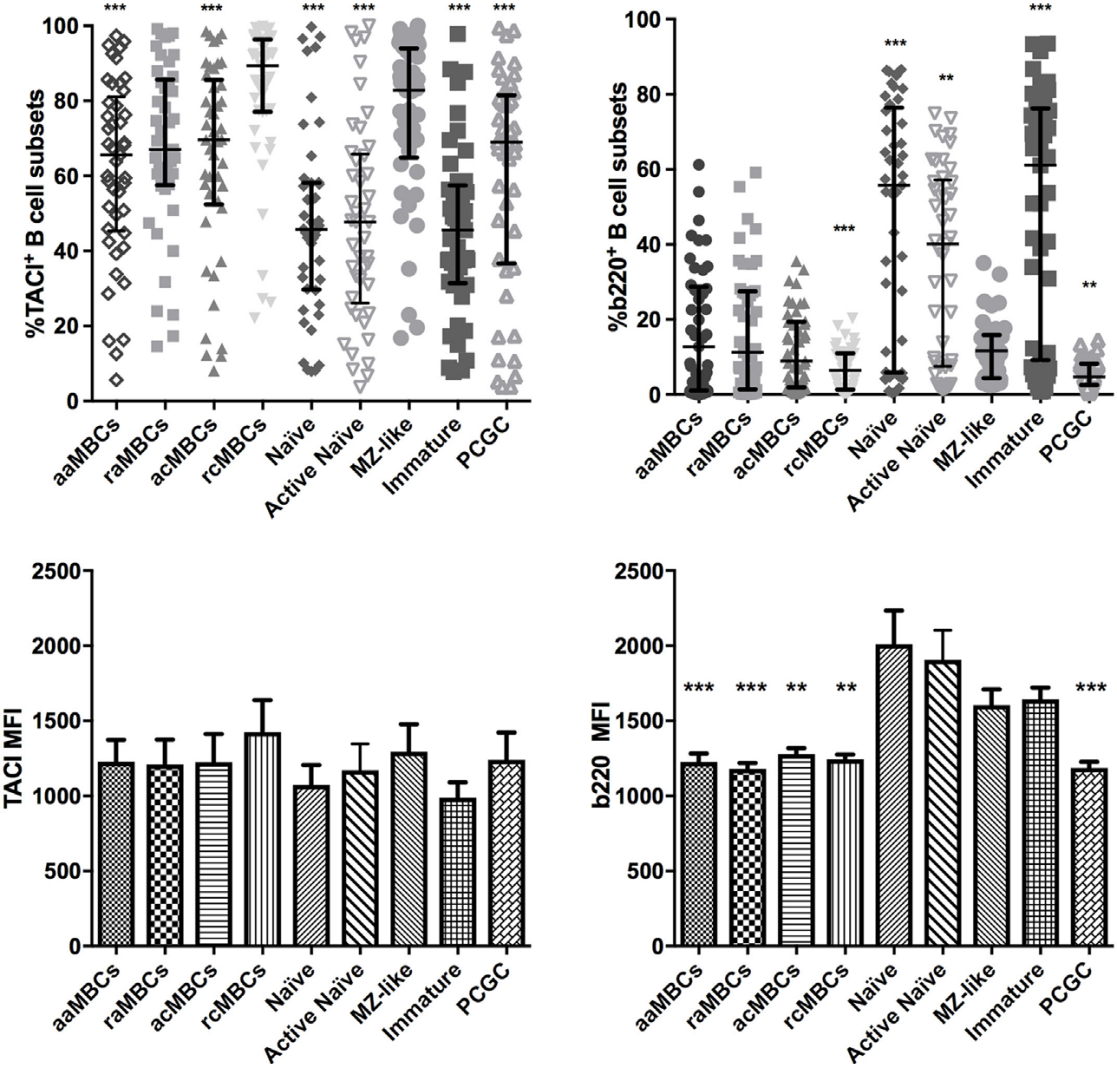
Distribution of expression of TACI and b220 B cell subpopulations in malaria-exposed individuals. Frequencies (%) expressed median and interquartile range of TACI-expressing B cells and b220-expressing B cells. Differences were assessed with the Friedman test plus Dunn’s *post hoc* test, comparing marginal zone (MZ)-like B cells versus every other B cell subset (*p < 0.05, **p < 0.01, ***p < 0.001). MBC, memory B cell; aaMBC, active atypical MBC; raMBC, resting atypical MBC; acMBC, active classical MBC; rcMBC, resting classical MBC; PCGC, plasmablast and germinal center cells.

Finally, we found that MZ-like B cells had increased expression of TACI. TACI is expressed mostly on MZ-like B cells, CD27^+^ MBCs, and plasma cells (31, 52). Here, TACI was mainly present on MZ-like B cells and rcMBCs and raMBCs, all expressing CD21 (Table 3; Table S2 in Supplementary Material; Figure 7). In addition, MZ-like B cells had lower frequencies of b220^+^ cells than the rest of the unswitched B cell subsets (Figure 7).

## Discussion

Our findings confirm that the circulating B cell compartment of pregnant women with frequent *Plasmodium* antigen exposure is dramatically altered, likely because of modifications in the frequency and phenotype of different B cell subsets due to chronic immune activation. Malaria exposure is associated with an increase of aaMBC and a decrease of MZ-like B cell frequencies in peripheral blood, as reported in HIV (51, 58) and previous malaria (20, 26) studies.

Individuals highly exposed to malaria had more PD1^+^, CD95^+^, CD80^+^, CD71^+^, and CD40^+^ aaMBC than malaria non-exposed individuals. Within the malaria-exposed, expression (% and/or MFI) of PD1 and CD95 was highest in aaMBCs, consistent with the higher expression of *PCPD1*, the signaling regulator of PD1, shown in aaMBCs (28). Individuals chronically infected with *Pf*, HIV, HBV, or HCV, have higher PD1 expression on T cells, a marker of both activation and immune suppression (2, 11, 25), and its blockade is also a target for immune therapies in HCV and melanomas (45, 59, 61, 63). To the best of our knowledge, this is the first time that increased PD1 expression has been described on B cell subsets in the context of chronic malaria exposure. In addition, increased expression of Fas/CD95 by aaMBC-like phenotypes has previously been found in HIV infection (13), rheumatoid arthritis disease (15) and active systemic lupus erythematosus (16). Malaria-related increase in CD95 and PD1 expression on aaMBCs could be responsible for their increased susceptibility to cell death (64). However, this seems unlikely, as aMBCs are found at high frequencies in malaria-exposed populations (3, 14, 21, 25, 60).

Increased CD40 expression associated to malaria exposure might favor aaMBC survival, as strong CD40L stimulus of GC cells has resulted in production of antiapoptotic proteins, providing survival signals rescuing the GC B cells from CD95-induced death (46). PD1 and CD95 expression in constantly activated B cells may be involved in mechanisms of immune inhibition rather than apoptosis (54) helping to establish tolerance to chronic infection and in agreement with previously detected higher expression of B cell survival genes in aaMBCs (28).

We also observed that malaria-exposed individuals had higher frequencies of CD80^+^ raMBC and CD80^+^ aaMBC than the non-exposed individuals but only raMBC had higher proportions of CD86^+^ cells in the exposed group. Triggering of CD80 inhibits proliferation by upregulating the expression of proapoptotic molecules and downregulating levels of antiapoptotic molecules, whereas CD86 boost B cell activity; both markers have been involved in apoptosis *via* the CD95/CD95L pathway (37, 55, 62). CD80 might interact with PD1L in a reverse signaling pathway that promotes B cell anergy, as seen on T cells (56). The mechanisms establishing the balance between immune inhibition and cell death are complex, but both mechanisms seem to play a role in tolerogenic responses to infection (54).

The proportion of IgG^+^ aaMBCs did not change with malaria exposure, whereas IgG^+^ raMBC and IgG^+^ rcMBC proportions increased with exposure levels. Chronic antigen exposure can cause downregulation of IgG surface levels after B cell receptor internalization *via* endocytosis in favor of transition to secretory Ig in activated B cells (9). We analyzed the expression of CD71 as a marker of B cell endocytosis (18) and observed that CD71^+^ aaMBCs were more frequent in exposed individuals, suggesting that aaMBCs recognize and internalize fewer antigens than other switched B cell subsets (18). The correlation between the number of cell surface CD71 molecules and the rate of cell proliferation is well known (47), as seen in numerous oncogenic cells (31, 39, 47–50). The increased frequencies of aaMBCs expressing CD71 in malaria-exposed pregnant women suggests that aaMBCs may proliferate at a higher rate in malaria-exposed individuals and is consistent with the overall higher frequencies of aaMBC in these individuals. Whether aaMBCs have increased capacity to proliferate or the higher CD71 expression on these cells need further studies.

Peripheral MZ-like B cells have been associated with T-independent immune responses and can undergo SHM (30, 31, 52). We confirmed decrease in circulating MZ-like B cell frequency (18, 26) and have shown, for the first time, TACI^+^ MZ-like B cell frequency reduction and higher CD95 expression with increasing malaria exposure. TACI deficiency impairs antibody responses to polysaccharide antigens, which may explain the increased vulnerability to infection with encapsulated organisms associated with malaria (29, 65). Also, the observed exposure-dependent reduced frequencies of TACI^+^ MZ-like B cells may jeopardize the production of long-lived antibody secreting cells (ASCs) at the expense of short-lived ASCs, as seen in malaria-exposed populations (66, 67) and is consistent with hypogammaglobulinemia, associated with deficiencies in TACI gene expression seen in common variable immunodeficiency disease (65). Also, highly exposed individuals have an increased frequency of IgG^+^ MZ-like B cells, although the difference was not statistically significant. This finding suggests that once TACI is activated after TLR-4 stimulation, there might be rapid T-independent antibody responses from peripheral MZ-like B cells that constitute an efficient first line of defense. Then, TACI-expressing peripheral MZ-like B cells upregulate CD95 and CD95L, and suppress inhibitors of CD95, which could result in apoptosis, preventing long-lasting activation (52). To the best of our knowledge, this is the first time that dual expression of CD95 and IgG on MZ-like B cells has been described in individuals with chronic antigen exposure. Although we propose that activated peripheral MZ-like B cells activate cell death (52), which explains part of the lower proportion of circulating MZ-like B cells in malaria exposed, we cannot rule out that part of this may be because active MZ-like B cells could be located in lymphoid organs (31). Also, non-antigen-specific cells, like active naïve B cells, have increased expression of CD95 and PD1 with malaria exposure, suggesting that chronic infections may alter the phenotype, but this finding requires future studies. We did not find differential expression of migration markers in B cell subsets associated to malaria exposure.

The study of the origin of atypical MBCs has been controversial, a study in malaria-exposed children reported that aMBCs and cMBCs may share similar rates of SHM, suggesting a shared origin (14) whereas other study suggested a different origin (68). We found differences in the expression of PD1, b220, CD86, CD62L, IgM, and IgG between acMBC and aaMBCs and we can speculate that acMBCs upregulate PD1, b220, CD86, and IgM and downregulate CD27 and CD62L to become aaMBC. But it also seems likely that a fraction of aaMBCS expressing b220 and/or IgM may have a GC-independent development pathway. This hypothesis would be consistent with recent observations where IgM^+^ aaMBC (IgD^−^CD21^−^CD27^−^) subsets appear to have two origins: a large fraction sharing mutation profiles similar to acMBCs (14), and a fraction with a clonal relationship similar to MZ-like B cells (34, 69). We propose that in a context of chronic malaria exposure, MZ-like B cells undergo GC-independent affinity maturation, by first upregulating b220 expression and then downregulating IgD, CD21, and CD27 to generate early IgM short-lived MBC (32, 42). This hypothesis accounts, in part, for the lower frequency of MZ-like B cells observed with increasing exposure and explains the similar IgM expression on active and resting aMBC found in all exposure levels. Recently published work has shown that *Pf*-specific IgM^+^ MBCs have a precursor relationship with IgM^+^ ASC, highlighting the importance of IgM^+^ B cells as effective responders to malaria rechallenge (33).

Our study had some limitations that may influence the results. We previously studied this cohort of pregnant women and found no differences in the proportion of aMBC and circulating MZ-like B cells between pregnant and non-pregnant malaria-exposed women (18). But pregnancy itself and sex could have had an effect on the expression of some markers between malaria-exposed pregnant women from PNG and non-exposed controls from Spain. We were incapable of providing non-exposed controls from PNG, as all women were somehow exposed to *Plasmodium* parasites; therefore exposure to other chronic infections in women from the Madang area could also have an effect. However, HIV prevalence is very low in the area. We were unable to provide absolute B cell counts, and frequencies were based on the total peripheral B cell population. Also, we analyzed total B cells and not *Plasmodium*-specific B cells, but recent work show good correlation with decreased AMA1-specific B cell frequencies and total B cells per milliliter of blood (69). Finally, the relatively small samples size may have had limited power to detect statistically significant differences between the low- and high-exposure groups.

We propose that malaria-associated expansion of active and resting aMBCs with increased expression of inhibitory PD1, CD95, and co-stimulatory CD80 in malaria-exposed women, coupled with increased CD71 and CD40 expression on aaMBCs, suggests that these cells are maintained in an anergic state (inhibitory profile) rather than going to apoptosis or cell death, helping to reduce the immune activation, and establishing a tolerogenic-like profile, as described on T cells (56, 70, 71). The expression of these markers seems to play a key role in establishing immune-homeostatic mechanisms that inhibit chronically activated B cells while simultaneously maintaining high B cell frequencies. This mechanism would keep the B cell activation threshold high enough to control infection but impaired enough to tolerate it and prevent systemic inflammation.

*Plasmodium* exposure decreased the frequencies of TACI expression and upregulated the frequencies of CD95 on circulating MZ-like B cells. TACI decreased frequency may result in a lack of capacity to generate long-lived antigen-specific IgG+ B cells, suggesting that increasing exposure leads to higher frequency of GC-independent MBC formation, favoring the rapid appearance of short-lived IgM^+^ ASC. The upregulation of CD95, the increased frequencies of IgG and b220 suggest that MZ-like B cells are being activated. This marks the importance of MZ-like B cells as targets for antimalarial therapies and vaccine development. However, functional assays should be performed to try to shed light on the exact mechanisms and role of aaMBCs and MZ-like B cells on chronic infections.

## Ethics Statement

Written informed consent was obtained from all study participants. This study was approved by the Medical Research Advisory Committee in PNG (MRAC 08.02) and by the Hospital Clinic of Barcelona Ethics Review Committee (Comitè Ètic d’Investigació Clínica), Spain.

## Author Contributions

IU performed the experiments, analyzed the data, and wrote the first draft of the manuscript. JC designed the flow cytometry panels and conceived the experimental design. PR obtained the preliminary data. MO, SH, and CD recruited and followed up volunteers. HR, PS, PR, GM, DB, and AJ processed lab samples. DB and AJ helped with performing the experiments. AB, IM, CM, SR, and CD contributed to the epidemiological study design and/or conduct. CD and GM supervised the experimental design and data analysis. JC, PR, GM, and CD interpreted the data. JC, PR, SR, GM, and CD participated in the writing up of the manuscript.

## Conflict of Interest Statement

The authors declare that the research was conducted in the absence of any commercial or financial relationships that could be construed as a potential conflict of interest.
