# Corrigendum: Chronic Exposure to Malaria Is Associated with Inhibitory and Activation Markers on Atypical Memory B Cells and Marginal Zone-Like B Cells

**DOI:** 10.3389/fimmu.2019.02427

**Published:** 2019-10-17

**Authors:** Itziar Ubillos, Joseph J. Campo, Pilar Requena, Maria Ome-Kaius, Sarah Hanieh, Honor Rose, Paula Samol, Diana Barrios, Alfons Jiménez, Azucena Bardají, Ivo Mueller, Clara Menéndez, Stephen Rogerson, Gemma Moncunill, Carlota Dobaño

**Affiliations:** ^1^ISGlobal, Barcelona Ctr. Int. Health Res. (CRESIB), Hospital Clínic-Universitat de Barcelona, Barcelona, Spain; ^2^Antigen Discovery Inc., Irvine, CA, United States; ^3^Facultad de Medicina, Universidad de Granada, Granada, Spain; ^4^Papua New Guinea Institute of Medical Research, Madang, Papua New Guinea; ^5^CIBER Epidemiology and Public Health (CIBERESP), Barcelona, Spain; ^6^Walter and Eliza Hall Institute of Medical Research, Parkville, VIC, Australia; ^7^University of Melbourne, Melbourne, VIC, Australia

**Keywords:** chronic infection, malaria, tolerance, B cells, host–malaria interaction

In the original article, we neglected to include the co-funder “FEDER funds/European Regional Development Fund (ERDF)” to “Instituto de Salud Carlos III (grant number PI14/01422)”.

A correction has been made to the **Funding** statement:

“This work was funded by the Instituto de Salud Carlos III (grant number PI14/01422), cofunded by FEDER funds/European Regional Development Fund (ERDF), the EU FP7 (PregVax, FP7/2007–2013 Grant 201588), the Ministerio de Economía y Competitividad (National R&D Internationalization Program, EUROSALUD 2008, Grant EUS2009-03560), and the Malaria in Pregnancy Consortium through the Bill & Melinda Gates Foundation (Grant 46099). GM was recipient of a Sara Borrell—ISCIII fellowship (CD010/00156). CD was supported by a fellowship from the Ministerio de Economía y Competitividad (Grant RYC-2008-02631) and was affiliate of the EU FP7 Network of Excellence EviMalaR. IU received funds from EviMalaR and the Agency for Management of University and Research Grants (AGAUR grant number 2014SGR991) for doctoral studies. ISGlobal is a member of the CERCA Programme, Generalitat de Catalunya.”

The authors apologize for this error and state that this does not change the scientific conclusions of the article in any way. The original article has been updated.

