# Supplementary material for: Chronic Exposure to Malaria Is Associated with Inhibitory and Activation Markers on Atypical Memory B Cells and Marginal Zone-Like B Cells
Source: Front Immunol. 2017 Aug 21;8:966. doi: 10.3389/fimmu.2017.00966 (PMC5573441; doi:10.3389/fimmu.2017.00966)

Supplementary Material

Chronic exposure to malaria is associated with inhibitory and activation markers on atypical memory B cells and marginal zone-like B cells

Ubillos I, Campo JJ, Requena P, Ome-Kaius M, Hanieh S, Rose H, Samol P, Barrios D, Jiménez A, Bardají A, Mueller I, Menéndez C, Rogerson S, Moncunill G, Dobaño C.

* **Correspondence**

E-mail: carlota.dobano@isglobal.org

Supplementary Figures and Tables

Supplementary Figure 1. Matrix of median fluorescence intensitiy (MFI) to PfMSP1$_{19}$, PfAMA1, PfEBA175, PvDBP, PvMSP1$_{19}$. Green dots represents antibody levels of individuals categorized as high malaria exposed and brown dots represents antibody levels of low malaria exposed individuals. Malaria exposure was based on breadth of IgG responses to *Plasmodium* antigens from 55 selected pregnant women.

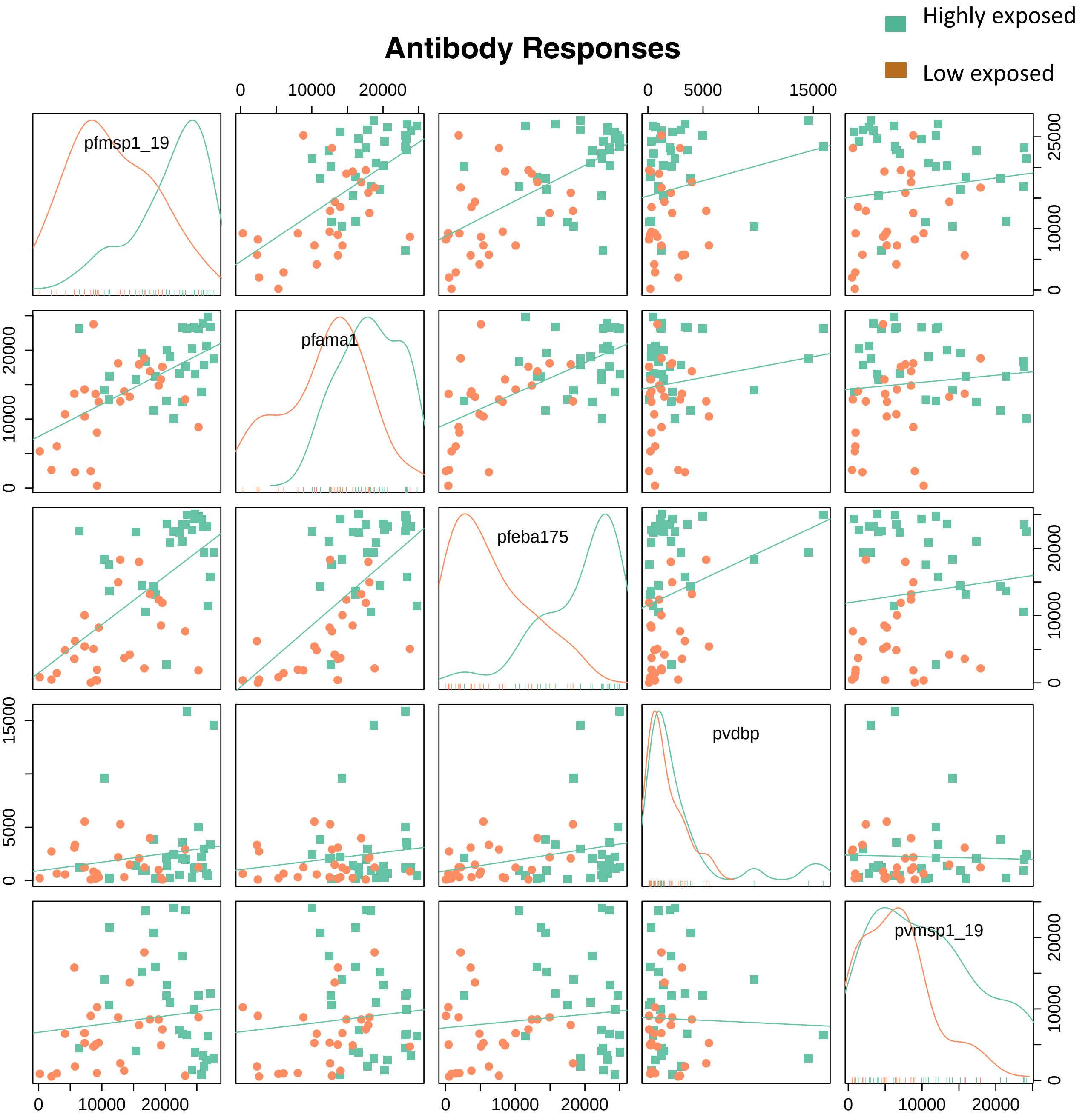

Supplementary Figure 2. Fluorescence-minus-one (FMO) controls for gating of positive events.

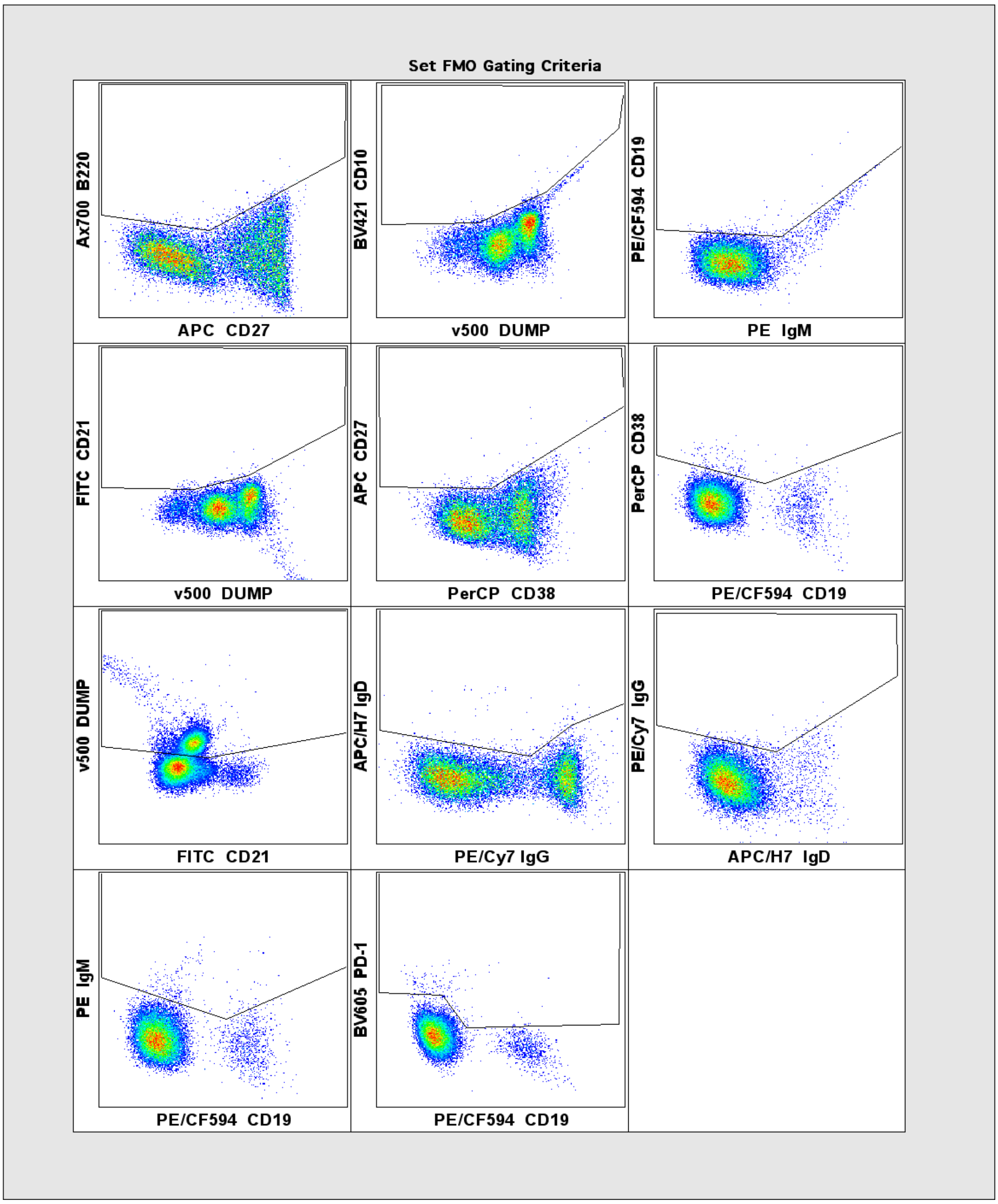

Supplementary Table 1. Frequencies of B cell subsets in malaria-infected and non-infected pregnant women. Means and 95% CI in active atypical MBCs (aaMBCs, CD3^{-}CD14^{-}CD16^{-}CD19^{+}CD10^{-}IgD^{-}CD21^{-}CD27^{-}); resting atypical MBCs (raMBCs, CD3^{-}CD14^{-}CD16^{-}CD19^{+}CD10^{-}IgD^{-}CD21^{+}CD27^{-}); active classical MBCs (acMBCs, CD3^{-}CD14^{-}CD16^{-}CD19^{+}CD10^{-}IgD^{-}CD21^{-}CD27^{+}); and resting classical MBCs (rcMBCs, CD3^{-}CD14^{-}CD16^{-}CD19^{+}CD10^{-}IgD^{-}CD21^{+}CD27^{+}); Plasmablasts and Germinal center cells (PCGC, CD3^{-}CD14^{-}CD16^{-}CD19^{+}CD10^{-}IgD^{-}CD38^{high}); Naïve (CD3^{-}CD14^{-}CD16^{-}CD19^{+}CD10^{-}IgD^{+} CD21^{+}CD27^{-}); Active naïve (CD3^{-}CD14^{-}CD16^{-}CD19^{+}CD10^{-}IgD^{+}CD21^{-}CD27^{-}); MZ-like B cells (CD3^{-}CD14^{-}CD16^{-}CD19^{+}CD10^{-}IgD^{+}CD21^{+}CD27^{+}IgM^{+}) and Immature B cells (CD3^{-}CD14^{-}CD16^{-}CD19^{+}CD10^{+}). *p-values from Wilcoxon rank-sum test adjusted for multiple testing by Benjamini and Hochberg method comparing malaria-exposed and non-exposed individuals.

B cell Subset	**% infected (N=8)**	**95% CI**	**% uninfected (N=47)**	**95% CI**	**P value**	**P value (Adj)**
aaMBCs	10.52	6.4-14.6	8.83	6.4-11.2	0.133	0.680
rcMBC	15.03	11.-19.0	15.87	13.-18.2	0.459	0.975
acMBCs	2.96	1.4-4.50	2.77	2.1-3.36	0.616	0.975
raMBCs	15.76	10.-21.0	18.06	15.-20.4	0.924	0.975
PCGC	3.23	1.3-5.11	4.17	3.1-5.21	0.567	0.975
Naive (n)	35.70	26.-45.2	30.99	27.-34.8	0.294	0.912
Active Naive (an)	4.67	2.9-6.36	3.00	2.4-3.58	0.015	0.126
Immature (Imm)	7.34	2.5-12.1	9.26	7.3-11.1	0.474	0.975
MZ-like	2.41	1.3-3.46	2.82	2.2-3.38	0.504	0.975
**B cell subset_marker**						
aaMBCs_IgG	66.1	56.2-76.0	56.2	49.5-62.8	0.233	0.656
aaMBCs_IgM	21.9	10.5-33.2	22.4	16.7-27.9	0.793	0.919
aaMBCs_b220	4.1	0.68-7.53	16.8	11.9-21.6	0.048	0.393
aaMBCs_PD1	19.6	8.08-31.0	19.8	15.1-24.3	0.924	0.957
rcMBC_IgG	53.3	46.3-60.1	46.9	40.7-52.9	0.328	0.723
rcMBC_IgM	21.9	14.6-29.2	25.2	19.7-30.5	0.924	0.957
rcMBC_b220	6.1	2.41-9.87	17.8	13.0-22.5	0.110	0.591
rcMBC_PD1	2.7	-0.3-5.65	1.5	0.92-2.13	0.720	0.893
acMBCs_IgG	74.6	66.9-82.1	64.1	58.7-69.4	0.174	0.656
acMBCs_IgM	11.8	8.85-14.6	14.8	12.1-17.3	0.703	0.880
acMBCs_b220	3.9	1.67-6.21	12.0	9.02-14.8	0.042	0.393
acMBCs_PD1	6.2	2.26-10.0	5.5	4.28-6.62	0.811	0.919
raMBCs_IgG	60.5	52.8-68.2	52.8	48.3-57.1	0.197	0.656
raMBCs_IgM	12.0	10.6-13.3	14.1	12.2-15.9	0.417	0.742
raMBCs_b220	3.0	1.52-4.52	6.8	5.24-8.40	0.121	0.603
raMBCs_PD1	3.3	-0.0-6.68	2.4	1.62-3.18	1.000	1.000
pcgc_IgG	20.1	4.72-35.4	11.3	8.67-13.9	0.174	0.656
pcgc_IgM	11.1	1.66-20.5	8.1	5.16-10.9	0.583	0.786
pcgc_b220	3.9	1.91-5.88	5.0	3.92-6.10	0.551	0.786
pcgc_PD1	5.2	-1.7-12.1	1.6	1.06-2.18	0.581	0.786
n_IgG	2.3	0.63-3.87	2.4	0.57-4.20	0.133	0.609

B cell Subset	% infected (N=8)	95% CI	% uninfected (N=47)	95% CI	P value	P value (Adj)
n_IgM	63.9	53.7-74.0	65.0	60.9-68.9	0.830	0.919
n_b220	32.7	13.7-51.6	49.5	40.5-58.3	0.127	0.603
n_PD1	1.4	-0.2-3.06	1.3	0.62-1.91	0.981	0.989
an_IgG	9.8	-3.4-23.0	5.7	3.32-8.04	0.886	0.947
an_IgM	53.7	39.5-67.8	55.0	51.1-58.8	0.867	0.935
an_b220	16.0	5.85-26.0	35.8	28.5-43.0	0.066	0.481
an_PD1	13.0	5.31-20.6	10.8	8.34-13.2	0.583	0.786
MZ-like_IgG	3.9	0.35-7.39	4.1	2.26-6.00	0.599	0.799
MZ-like_b220	4.2	0.97-7.45	4.3	2.04-6.53	0.535	0.786
MZ-like_PD1	14.9	6.21-23.5	14.4	12.0-16.8	0.830	0.919
Imm_IgG	15.5	8.32-22.6	18.9	15.0-22.7	0.535	0.786
Imm_IgM	63.5	56.7-70.3	54.2	50.0-58.2	0.081	0.481
Imm_b220	36.4	13.7-59.1	52.0	42.8-61.1	0.159	0.656
Imm_PD1	2.8	0.28-5.31	1.8	1.22-2.35	0.322	0.723
aaMBCs_CD40	64.3	43.3-85.1	72.9	66.4-79.3	0.189	0.656
aaMBCs_CD95	89.5	83.2-95.7	82.9	79.3-86.3	0.139	0.615
aaMBCs_TACI	72.4	50.0-94.8	65.0	58.1-71.9	0.328	0.723
aaMBCs_CD150	15.3	0.39-30.2	14.5	11.0-17.9	0.667	0.853
rcMBC_CD40	92.4	85.0-99.8	95.9	94.4-97.2	0.377	0.742
rcMBC_CD95	76.4	63.8-88.9	61.6	55.7-67.4	0.030	0.336
rcMBC_TACI	75.6	52.0-99.1	69.8	63.7-75.9	0.252	0.677
rcMBC_CD150	17.8	1.54-34.1	12.7	9.31-16.1	0.756	0.919
acMBCs_CD40	66.4	45.9-86.9	72.1	67.0-77.2	0.583	0.786
acMBCs_CD95	91.6	87.0-96.1	83.4	80.7-85.9	0.009	0.164
acMBCs_TACI	76.7	53.4-99.8	67.0	59.7-74.2	0.145	0.621
acMBCs_CD150	16.3	0.26-32.4	15.6	12.2-18.9	0.305	0.723
raMBCs_CD40	81.1	69.5-92.6	86.1	83.0-89.1	0.474	0.763
raMBCs_CD95	87.6	79.5-95.5	74.8	71.3-78.2	0.003	0.115
raMBCs_TACI	84.4	62.7-106.	83.3	77.9-88.6	0.283	0.701
raMBCs_CD150	13.2	-1.2-27.5	10.1	6.95-13.2	0.830	0.919
pcgc_CD40	26.8	9.18-44.5	26.8	21.7-31.8	0.650	0.840
pcgc_CD95	70.2	40.7-99.6	85.0	77.4-92.5	0.445	0.756
pcgc_TACI	78.7	54.7-102.	65.2	56.2-74.1	0.181	0.656
pcgc_CD150	59.3	34.9-83.5	51.4	45.7-57.0	0.504	0.786
n_CD40	94.0	88.7-99.1	96.3	95.1-97.5	0.535	0.786
n_CD95	38.8	18.2-59.3	14.8	9.78-19.8	0.002	0.115
n_TACI	59.6	32.1-86.9	48.6	42.1-55.1	0.283	0.701
n_CD150	46.5	24.3-68.5	35.1	28.7-41.3	0.233	0.656
an_CD40	72.6	57.4-87.6	80.7	76.9-84.3	0.206	0.656
an_CD95	58.9	43.5-74.1	43.8	38.6-49.0	0.045	0.393
an_TACI	62.0	36.8-87.1	49.8	42.3-57.1	0.242	0.667
an_CD150	26.9	13.6-40.1	22.7	18.3-26.9	0.459	0.763
MZ-like_CD95	70.9	48.0-93.8	41.5	34.2-48.6	0.011	0.164
MZ-like_TACI	70.0	49.7-90.3	75.6	69.6-81.4	0.535	0.786
MZ-like_CD150	61.0	34.0-88.0	32.3	24.3-40.3	0.009	0.164
Imm_CD40	86.4	75.2-97.4	93.6	90.9-96.1	0.011	0.164

B cell Subset	% infected (N=8)	95% CI	% uninfected (N=47)	95% CI	P value	P value (Adj)
Imm_CD95	40.0	22.7-57.3	22.3	16.4-28.1	0.017	0.211
Imm_TACI	56.4	34.5-78.3	46.0	40.1-51.8	0.206	0.656
Imm_CD150	28.7	11.3-46.1	19.7	14.6-24.7	0.127	0.603
aaMBCs_CCR3	17.5	0.45-34.4	17.1	10.8-23.3	0.830	0.919
aaMBCs_CxCR3	5.5	-2.2-13.1	2.5	1.53-3.53	0.981	0.989
aaMBCs_CD71	55.5	34.1-76.8	46.1	39.6-52.5	0.206	0.656
aaMBCs_CD62l	13.1	2.44-23.8	15.8	12.3-19.3	0.305	0.723
rcMBC_CCR3	22.9	-3.0-48.7	12.7	5.44-19.9	0.100	0.561
rcMBC_CxCR3	12.7	-10.-36.0	3.0	2.04-3.99	0.756	0.919
rcMBC_CD71	41.0	18.2-63.6	34.0	27.0-40.9	0.390	0.742
rcMBC_CD62l	33.3	14.7-51.8	31.4	26.2-36.4	0.924	0.957
acMBCs_CCR3	20.4	-0.0-40.8	14.4	8.49-20.2	0.474	0.763
acMBCs_CxCR3	7.1	-5.8-19.9	2.3	1.39-3.30	0.782	0.919
acMBCs_CD71	48.3	22.5-74.0	45.3	38.0-52.5	0.793	0.919
acMBCs_CD62l	27.4	12.9-41.8	31.7	27.5-35.9	0.377	0.742
raMBCs_CCR3	22.0	-1.7-45.6	12.2	5.64-18.8	0.073	0.481
raMBCs_CxCR3	10.8	-10.-31.8	2.2	1.38-2.92	0.830	0.919
raMBCs_CD71	47.0	20.6-73.3	37.8	30.1-45.4	0.390	0.742
raMBCs_CD62l	43.6	24.9-62.3	42.1	36.5-47.5	0.811	0.919
pcgc_CCR3	25.3	7.81-42.7	20.8	15.2-26.3	0.417	0.742
pcgc_CxCR3	4.4	-1.7-10.5	2.4	1.47-3.33	0.633	0.826
pcgc_CD71	68.7	43.9-93.4	65.9	58.5-73.2	0.431	0.742
pcgc_CD62l	19.7	3.23-36.2	18.8	15.0-22.4	0.583	0.786
n_CCR3	18.7	-6.6-43.9	8.9	3.86-14.0	0.077	0.481
n_CxCR3	10.6	-9.5-30.7	1.3	0.71-1.94	0.403	0.742
n_CD71	23.5	-1.2-48.2	13.1	5.55-20.6	0.081	0.481
n_CD62l	35.8	17.2-54.2	40.5	34.0-46.9	0.519	0.786
an_CCR3	21.3	-2.3-44.9	11.9	4.93-18.8	0.474	0.763
an_CxCR3	5.9	-3.7-15.6	3.0	2.05-3.99	0.429	0.742
an_CD71	21.2	1.03-41.3	17.5	11.0-23.9	0.340	0.723
an_CD62l	10.2	2.41-18.0	11.8	7.61-15.9	0.981	0.989
MZ-like_CCR3	25.6	2.64-48.5	12.3	5.32-19.2	0.001	0.115
MZ-like_CxCR3	11.6	-7.5-30.6	3.0	2.04-4.01	0.340	0.723
MZ-like_CD71	24.0	1.30-46.6	13.6	8.04-19.0	0.223	0.656
MZ-like_CD62l	30.9	8.44-53.3	32.0	27.0-37.0	0.431	0.742
Imm_CCR3	19.0	-2.3-40.3	10.6	4.79-16.4	0.233	0.656
Imm_CxCR3	8.6	-8.4-25.6	1.8	1.15-2.51	0.383	0.742
Imm_CD71	25.1	1.75-48.4	16.9	10.4-23.4	0.685	0.867
Imm_CD62l	22.4	9.58-35.2	30.5	25.0-36.0	0.262	0.677
aaMBCs_CD80	5.6	0.97-10.1	9.7	7.30-12.0	0.220	0.656
aaMBCs_CD86	18.0	10.3-25.5	37.4	32.5-42.1	0.004	0.115
rcMBC_CD80	8.3	4.09-12.4	10.4	8.39-12.3	0.609	0.803
rcMBC_CD86	17.1	-7.0-41.1	19.8	15.2-24.4	0.257	0.677
acMBCs_CD80	7.9	-0.9-16.6	8.0	5.83-10.2	0.926	0.957
acMBCs_CD86	18.0	11.2-24.8	31.4	27.2-35.6	0.013	0.172

B cell Subset	% infected (N=8)	95% CI	% uninfected (N=47)	95% CI	P value	P value (Adj)
raMBCs_CD80	7.6	-0.7-15.9	5.7	4.33-6.97	0.545	0.786
raMBCs_CD86	17.7	-2.5-37.9	18.4	14.7-22.1	0.344	0.723
pcgc_CD80	4.3	-0.9-9.57	5.9	4.40-7.45	0.576	0.786
pcgc_CD86	78.1	50.7-105.	89.1	86.5-91.6	0.209	0.656
n_CD80	0.6	0.02-1.20	1.2	0.71-1.59	0.328	0.723
n_CD86	11.7	-11.-34.8	6.7	3.24-10.2	0.864	0.935
an_CD80	1.4	-0.4-3.12	4.0	2.71-5.26	0.039	0.393
an_CD86	15.9	-13.-45.3	19.6	15.0-24.2	0.052	0.406
MZ-like_CD80	3.4	-0.8-7.66	2.8	2.22-3.37	0.864	0.935
MZ-like_CD86	18.5	-17.-54.5	11.7	8.27-15.2	0.376	0.742
Imm_CD80	5.5	-5.3-16.2	5.2	3.51-6.92	0.429	0.742
Imm_CD86	15.4	-5.7-36.5	14.5	10.3-18.5	0.545	0.786

Supplementary Figure 3. Frequencies of active atypical memory B cells among malaria exposed and non-exposed individuals. Beeswarm plots show active atypical MBCs frequencies categorized by malaria exposure and expressing activation, co-stimulation, inhibition (IgG, IgM, b220, CD40, CD150, CD80, CD86, PD1, CD95 and TACI) and migration markers (CD71, CCR3, CxCR3, CD62L). Lines and whiskers represent median and interquartile range, respectively. Differences between each exposure category were assessed with two-sample Wilcoxon rank-sum test *p<0.05, **p<0.01, ***p<0.001.

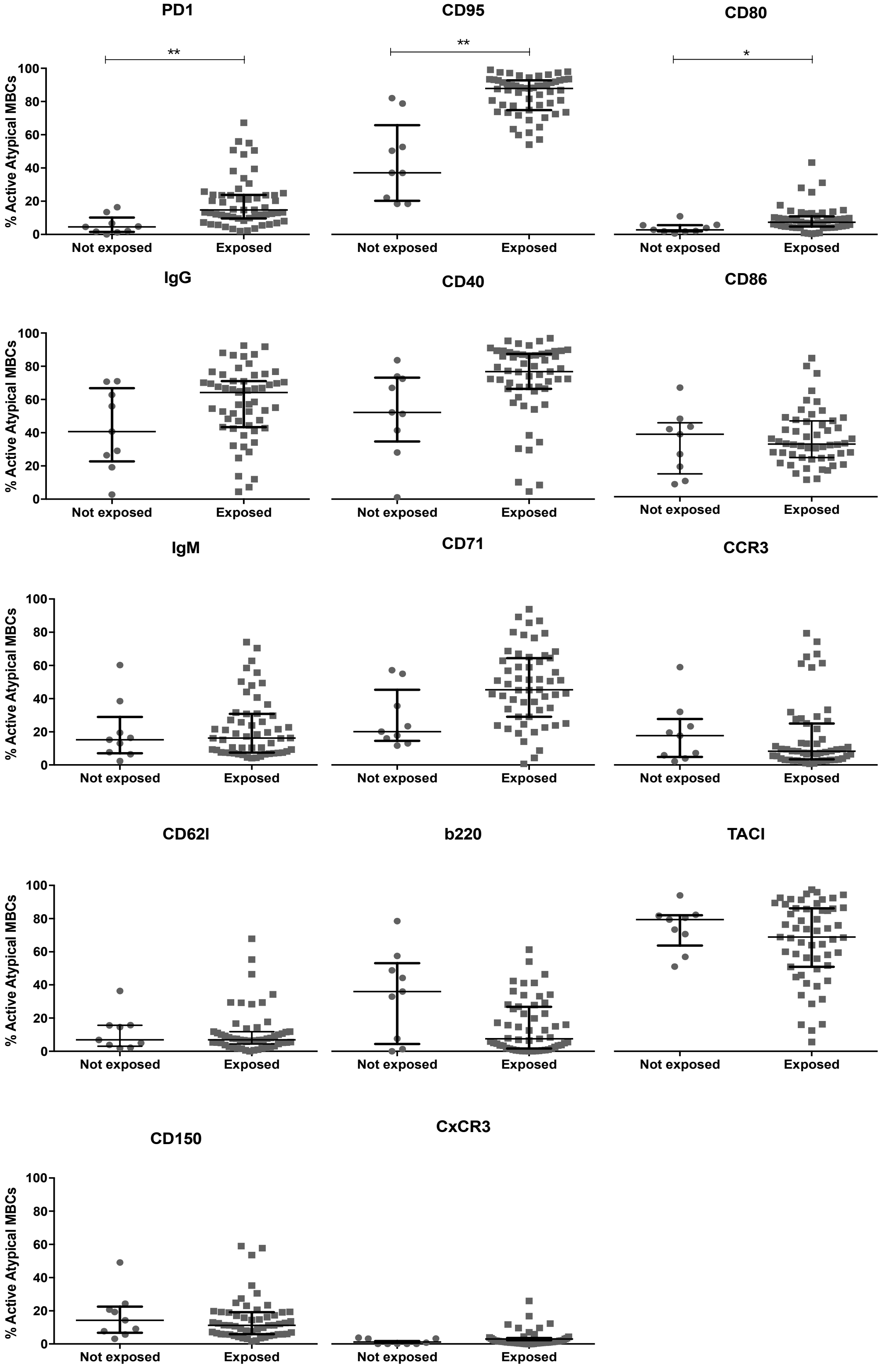

Supplementary Table 2. Frequencies in malaria-exposed and non-exposed. Means and 95% CI in **A)** Active Classical memory B cells (MBCs) ($CD19^{+}CD10^{-}IgD^{-}CD21^{-}CD27^{+}$); Resting Classical MBCs ($CD19^{+}CD10^{-}IgD^{-}CD21^{+}CD27^{+}$); PGCG indicates Plasmablasts and Germinal center cells ($CD19^{+}CD10^{-}IgD^{-}CD38^{high}$ and **B)** Naïve ($CD19^{+}$ $CD10^{-}IgD^{+}CD21^{+}CD27^{-}$); Active naïve ($CD19^{+}$ $CD10^{-}IgD^{+}CD21^{-}CD27^{-}$) and Immature ($CD19^{+}CD10^{+}$). *p-values from Wilcoxon rank-sum test adjusted for multiple testing by Benjamini and Hochberg method comparing malaria-exposed and non-exposed individuals.

A.

	**Active Classical MBC** mean (95% CI)			**Resting Classical MBC** mean (95% CI)			**PCGC** mean (95% CI)		
	**Not Exposed**	**Exposed**	**p-value**	**Not Exposed**	**Exposed**	**p-value**	**Not Exposed**	**Exposed**	**p-value**
**IgG**	45.58 (30.5-60.6)	65.61 (60.8-70.3)	0.027	30.59 (20.5-40.5)	53.91 (49.9-57.8)	0.006	11.73 (5.8-17.6)	12.58 (9.6-15.5)	0.915
**IgM**	27.27 (14.9-39.6)	14.32 (12-16.5)	0.052	40.6 (27.9-53.2)	13.81 (12.2-15.3)	0.002	18.91 (5.1-32.6)	8.51 (5.7-11.2)	0.015
**b220**	12.89 (2.8-22.9)	10.78 (8.1-13.4)	0.923	4.23 (1.1-7.3)	6.26 (4.8-7.6)	0.388	4.65 (-0.3-9.6)	4.85 (3.8-5.8)	0.346
**PD1**	5.38 (1.2-9.5)	5.55 (4.4-6.6)	0.72	1.28 (0.3-2.1)	2.53 (1.7-3.3)	0.395	0.8 (-0.1-1.7)	2.14 (1.1-3.1)	0.189
**CD40**	50.55 (34.8-66.2)	71.3 (66.3-76.2)	0.015	78.52 (66.3-90.7)	85.41 (82.4-88.3)	0.224	13.83 (8-19.6)	26.78 (22-31.5)	0.113
**CD95**	72.62 (64-81.1)	84.56 (82.1-86.9)	0.027	43.87 (28.5-59.1)	76.64 (73.3-79.9)	0.002	81.17 (61.2-99.1)	82.88 (75.4-90.2)	0.164
**TACI**	83.25 (74.4-92)	68.39 (61.6-75.1)	0.217	95 (91.5-98.4)	83.44 (78.2-88.6)	0.109	78.34 (67-89.6)	67.13 (58.9-75.3)	0.693
**CD150**	20.5 (9.7-31.2)	15.72 (12.3-19.1)	0.353	10.45 (-1.3-22.2)	10.55 (7.3-13.7)	0.373	52.27 (35.7-68.7)	52.51 (46.8-58.1)	0.905
**CCr3**	18.56 (-1.8-38.9)	15.27 (9.6-20.8)	0.373	13.55 (-8.1-35.2)	13.66 (7.3-19.9)	0.69	34.52 (15.9-53)	21.47 (16.3-26.6)	0.164
**CxCR3**	1.74 (-0.1-3.6)	3.03 (1.2-4.8)	0.702	1.38 (0.5-2.2)	3.41 (0.7-6)	0.656	1.3 (0-2.6)	2.69 (1.6-3.7)	0.309
**CD71**	45.55 (26.1-64.9)	45.75 (38.9-52.5)	0.977	26.45 (6.7-46.2)	39.13 (31.9-46.3)	0.253	76.74 (61.6-91.8)	66.29 (59.4-73.1)	0.388
**CD62l**	23.63 (15.9-31.3)	31.1 (27.1-35)	0.224	30.16 (20.8-39.4)	42.29 (37.1-47.4)	0.086	12.16 (5.1-19.1)	18.9 (15.2-22.5)	0.33
**CD80**	3.86 (1.2-6.4)	8 (5.9-10)	0.149	2.94 (1.7-4.1)	5.84 (4.5-7.1)	0.097	78.56 (62.1-94.9)	88.02 (85-91)	0.339
**CD86**	26.8 (15.6-37.9)	30.13 (26.1-34)	0.744	9.36 (1.6-17)	18.34 (14.7-21.9)	0.015	78.56 (62.1-94.9)	88.02 (85-91)	0.149

B.

	Naive mean (95% CI)			Active naive mean (95% CI)			Immature mean (95% CI)		
	**Not Exposed**	**Exposed**	**p-value**	**Not Exposed**	**Exposed**	**p-value**	**Not Exposed**	**Exposed**	**p-value**
**IgG**	0.58 (0-1.2)	2.36 (0.8-3.9)	0.052	2.08 (0.4-3.7)	6.27 (3.7-8.8)	0.164	5.81 (1.5-10.1)	18.39 (15-21.7)	0.015
**IgM**	62.41 (50.2-74.5)	64.79 (61.2-68.3)	0.721	65.39 (53.4-77.3)	54.78 (51.1-58.4)	0.149	68.2 (64.1-72.2)	55.54 (51.8-59.2)	0.051
**b220**	54.25 (30.2-78.2)	47.02 (38.9-55)	0.692	57.91 (31.3-84.4)	32.94 (26.3-39.5)	0.113	55.05 (27.7-82.3)	49.74 (41.4-58)	0.695
**PD1**	0.41 (0-0.7)	1.29 (0.7-1.8)	0.217	1.35 (0.6-2.1)	11.1 3(8.8-13.4)	0.002	0.91 (0.2-1.5)	1.93 (1.3-2.5)	0.247
**CD40**	92.19 (83.3-99.9)	95.99 (94.8-97.1)	0.697	77.72 (62.7-92.6)	79.5 (75.8-83.1)	0.855	83.76 (69.1-98.3)	92.5 (89.8-95.1)	0.182
**CD95**	10.69 (-2.3-23.7)	18.31 (12.8-23.7)	0.131	21.45 (5.8-37)	46.03 (41-50.9)	0.014	13.14 (-0.4-26.7)	24.88 (19.2-30.5)	0.108
**TACI**	65.69 (46.4-84.9)	50.2 (43.7-56.6)	0.161	70.34 (53.5-87.1)	51.53 (44.4-58.5)	0.113	57.6 (38-77.1)	47.52 (41.8-53.2)	0.329
**CD150**	13.9 (0.3-27.4)	36.72 (30.6-42.7)	0.014	12.62 (2.9-22.3)	23.29 (19.3-27.2)	0.087	8.91 (-3.5-21.3)	21.03 (16.2-25.8)	0.018
**CCr3**	11.54 (-10.3-33.4)	10.35 (5-15.6)	0.371	9.49 (-6.1-25.1)	13.27 (6.6-19.8)	0.888	11.68 (-9-32.4)	11.82 (6.2-17.4)	0.913
**CxCR3**	1.14 (0.2-2)	2.67 (0.1-5.2)	0.88	3.17 (1.2-5)	3.44 (2-4.8)	0.7	1.65 (0.3-2.9)	2.82 (0.6-4.9)	0.915
**CD71**	12.4 (-9.9-34.7)	14.62 (7.5-21.7)	0.132	8.83 (-3.4-21.1)	18.04 (12-24)	0.105	14.62 (-6-35.3)	18.12 (11.9-24.3)	0.305
**CD62l**	26.87 (13.2-40.4)	39.79 (33.8-45.7)	0.331	11.37 (2.9-19.8)	11.56 (7.8-15.2)	0.897	11.97 (3.4-20.5)	29.33 (24.3-34.3)	0.05
**CD80**	0.58 (0.3-0.8)	1.1 (0.7-1.4)	0.492	4.4 (0.9-7.8)	3.73 (2.5-4.9)	0.523	2.02 (0.8-3.2)	5.24 (3.5-6.9)	0.164
**CD86**	7.4 (-0.2-15)	7.21 (3.7-10.6)	0.915	12.23 (4.2-20.1)	19.28 (14.7-23.8)	0.164	9.5 (3-15.9)	14.56 (10.6-18.4)	0.641

Supplementary Figure 4. Frequencies of active and resting classical memory B cells (MBCs). Beeswarm plots show classical MBCS expressing CD95 and CD40 among malaria exposure categories. Lines and whiskers represent median and interquartile range, respectively. Differences were asses with Kurskal-Walllis test plus Dunn *post hoc* test comparing each exposure category p<0.05, **p<0.01, ***p<0.001. acMBCs indicates active classical MBCs, and rcMBCs resting classical MBCs.

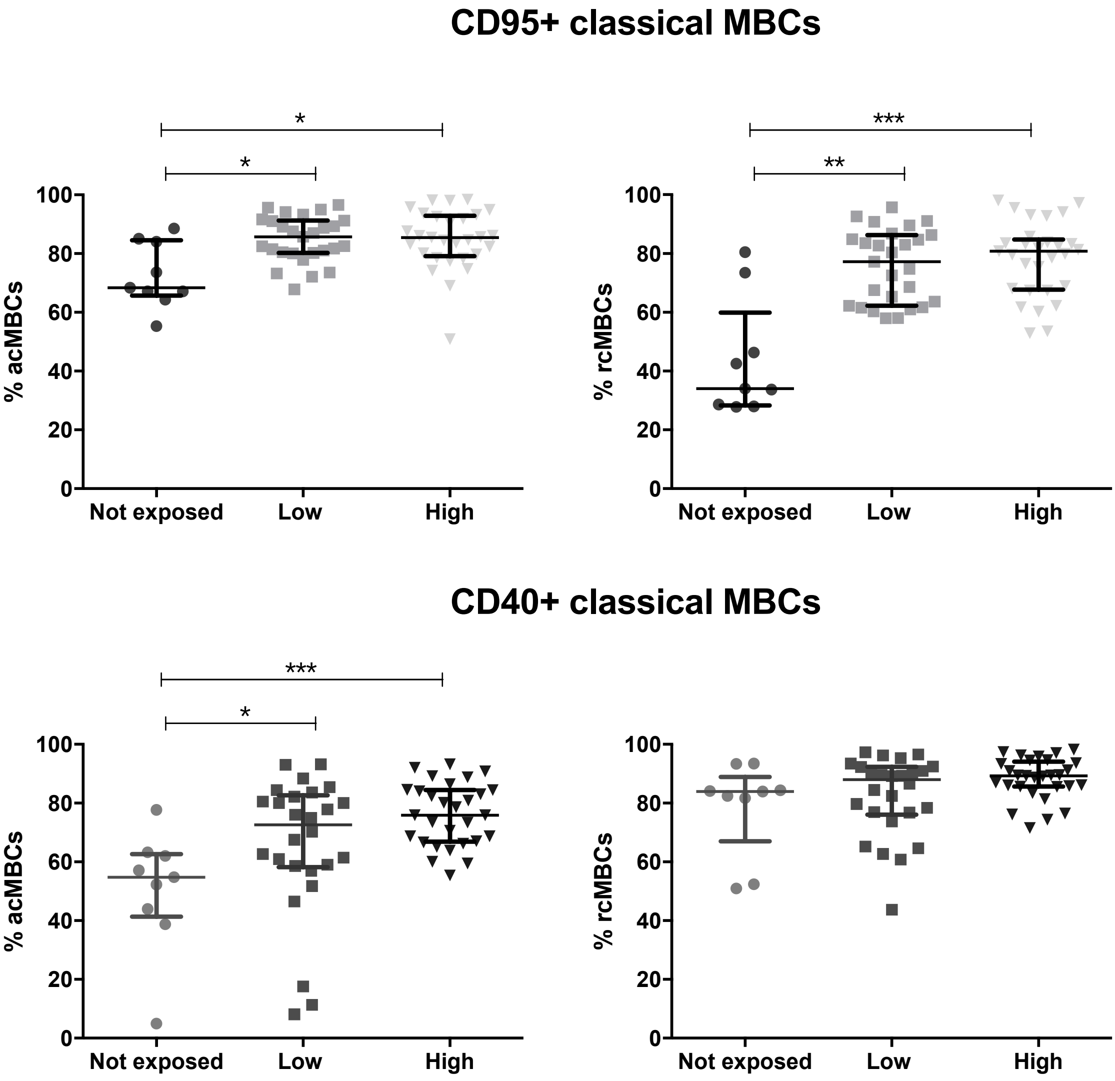

Supplementary Figure 5. Frequencies and magnitude of expression (MFI) of markers in aaMBCs and acMBCs. A) Differential frequencies of expression and B) Differential magnitude (MFI levels) between aaMBCs and acMBCs. Differences were assessed by Wilcoxon rank sum paired test. $p<0.05$, **$p<0.01$, ***$p<0.001$. acMBCs indicates active classical MBCs and acMBCs indicates active classical MBCs. Only significant differences are shown.

A.

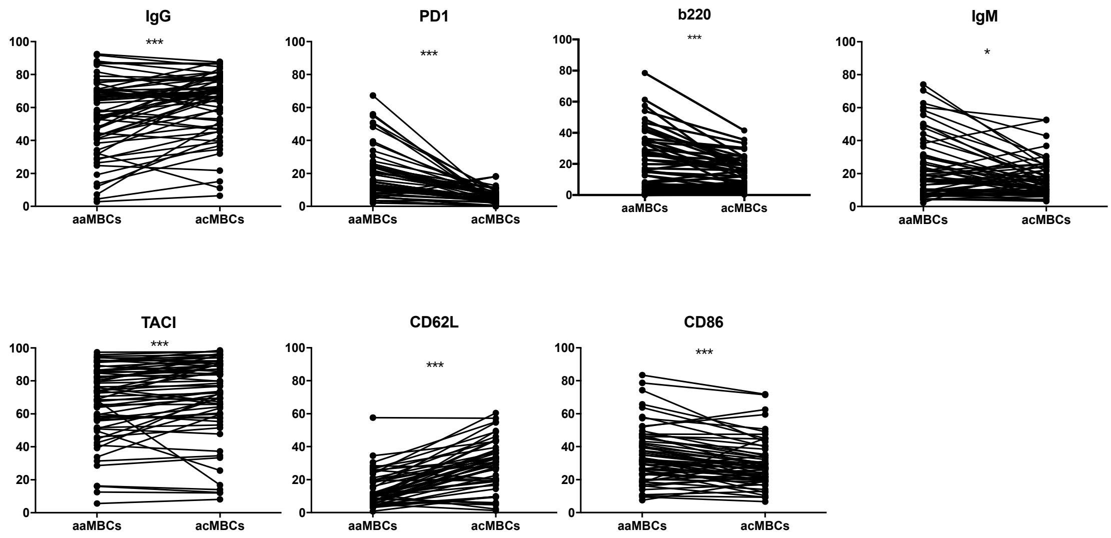

B.

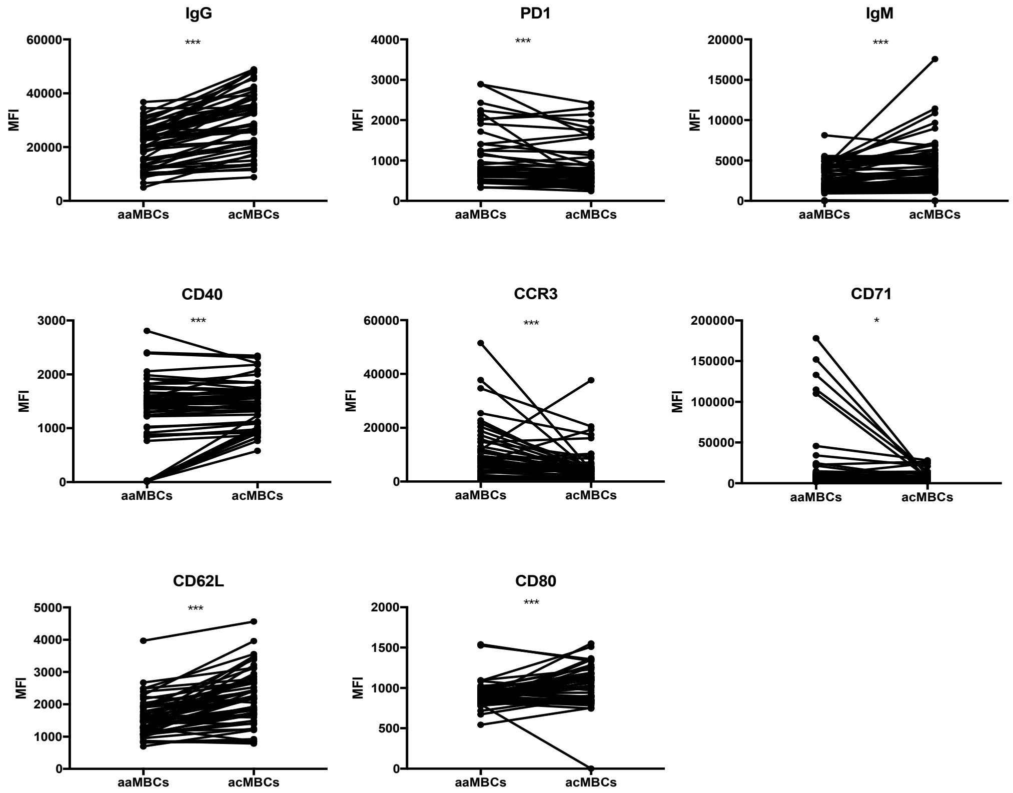

Supplement: Supplementary file 1 [file Presentation_1.pdf]
